# *PvE1* plays an essential role in regulating photoperiod sensitivity and flowering time in common bean

**DOI:** 10.1093/hr/uhag021

**Published:** 2026-01-22

**Authors:** Ana M González, Ana M Pesqueira, Rocío Fonseca, Sandra Bretones, Fernando J Yuste-Lisbona, Rafael Lozano, Marta Santalla

**Affiliations:** Grupo Genética del Desarrollo de Plantas, Misión Biológica de Galicia-CSIC, PO Box 28, 36080 Pontevedra, Spain; Grupo Genética del Desarrollo de Plantas, Misión Biológica de Galicia-CSIC, PO Box 28, 36080 Pontevedra, Spain; Centro de Investigación en Biotecnología Agroalimentaria (CIAIMBITAL), Universidad de Almería, 04120 Almería, Spain; Centro de Investigación en Biotecnología Agroalimentaria (CIAIMBITAL), Universidad de Almería, 04120 Almería, Spain; Centro de Investigación en Biotecnología Agroalimentaria (CIAIMBITAL), Universidad de Almería, 04120 Almería, Spain; Centro de Investigación en Biotecnología Agroalimentaria (CIAIMBITAL), Universidad de Almería, 04120 Almería, Spain; Grupo Genética del Desarrollo de Plantas, Misión Biológica de Galicia-CSIC, PO Box 28, 36080 Pontevedra, Spain

## Abstract

Flowering time is a critical trait in common bean (*Phaseolus vulgaris*), influencing yield stability and geographical adaptation. While *PvCOL2* and *PvPHYA3* are known regulators under long-day (LD) conditions, we identified a third major locus through fine-mapping of the QTL *DTF9.4/DTF9.5*. Within this region, *PvE1* (*Phvul.009G204600*) emerged as a strong candidate, sharing sequence homology with the soybean *E1* gene and acting as a transcriptional repressor of flowering. A naturally occurring 34-bp deletion in its 3′ UTR (*e1-del*) was associated with early flowering and reduced photoperiod sensitivity. Expression analysis revealed that *PvE1* displays a circadian rhythm under LD conditions, with a bimodal pattern peaking in the morning and early evening, resembling that reported for soybean *E1*. Phenotypic analyses of near-isogenic lines (NILs) confirmed that *PvE1* delays flowering specifically under LD and also influences plant architecture, as *e1* genotypes exhibited reduced plant height and node number. Functional dissection revealed that *PvE1* and *PvCOL2* act in partially redundant pathways to repress *PvFT* gene expression, with evidence of functional interaction. This regulatory module resembles that in soybean but shows species-specific divergence likely shaped by separate evolutionary paths. Genetic diversity analysis identified two rare *PvE1* alleles, *e1-del* and *e1-fs*, both associated with earlier flowering when combined with *col2* mutations, indicating additive effects and reduced photoperiod sensitivity. Although functional validation by transformation was not performed, the use of NILs provides robust genetic evidence of *PvE1* activity. Together, these findings establish *PvE1* as a conserved legume-specific floral repressor in common bean, with novel allelic variants that can be exploited to develop early-flowering, photoperiod-insensitive cultivars adapted to temperate and high-latitude regions.

## Introduction

Common bean (*Phaseolus vulgaris* L.) is an important legume crop with a long history of domestication in southern Mexico and the Andean highlands approximately 8000 years ago [1–4]. Derived from its wild tropical progenitor, the common bean exhibits substantial photoperiod sensitivity, requiring short-day (SD) conditions to flower [5, 6]. Through natural and artificial selection, common bean has evolved reduced photoperiod sensitivity, enabling it to adapt to both SD and long-day (LD) environments, allowing its cultivation from tropical regions to high-latitude zones (>40°N). Understanding the genetic basis of this variation is crucial for elucidating the domestication history of the crop and for identifying genes involved in photoperiodic flowering regulation, thereby aiding in the development of more productive varieties [1, 7, 8].

In *Arabidopsis thaliana*, the *CONSTANS (CO)–FLOWERING LOCUS T (FT)* module is a well-established pathway for photoperiodic flowering induction, where *CO* integrates circadian and lightsignals to activate *FT* expression under LD conditions, thereby promoting flowering [9, 10]. However, this central regulatory mechanism is not conserved in legumes, which exhibit a more diverse array of flowering-time pathways. Studies in legumes, including predominantly SD species, such as soybean, and LD species, such as pea (*Pisum sativum*), have primarily focused on the circadian clock and *FT*-like genes, revealing substantial variation in photoperiodic regulation across species [11–14]. Notably, the *CO* gene itself appears to be absent or non-functional in many legumes [15, 16].

In temperate LD legumes like *Medicago truncatula*, *CO* plays only a minor role in photoperiod measurement, with *CO*-independent mechanisms likely playing a more prominent regulatory function [16]. Furthermore, *CONSTANS-LIKE (COL)* genes in soybean, such as *COL1a/COL1b* and *COL2a/COL2b*, act as floral repressors under LD conditions, in contrast to CO's role in Arabidopsis [17–19]. A similar pattern is seen in common bean, where *PvCOL2* functions as a repressor of flowering under LD by downregulating *FT* gene expression [20]. These findings indicate that legumes employ distinct, and in some cases inverted, regulatory modules governing photoperiodic flowering.

In addition to *COL* genes, phytochromes—particularly *PHYA* and *PHYB*—are key components of the photoperiod response in legumes. In soybean, among four *PHYA*, two *PHYB*, and two *PHYE* paralogs, *PHYA2* and *PHYA3* have been identified as principal photoperiod receptors promoting delayed flowering under LD conditions in a *LUX ARRHYTHMO* (*LUX*)-dependent manner [21, 22]. This *PHYA*-mediated regulation of flowering is conserved in other legumes such as *Medicago* and pea, where *PHYA* plays a dominant role in the photoperiod response [23, 24]. In common bean, *Photoperiod* (*Ppd*)/*PvPHYA3* has been reported as the major photoperiod sensitivity locus, with early-flowering alleles under LD conditions linked to increased expression of several *PvFT* genes, suggesting that photoperiod adaptation involves modulation of *FT* activation pathways [25].

A central component of the soybean photoperiod pathway is the legume-specific B3 domain transcription factor *E1*, which represses *FT*-like genes (*GmFT2a* and *GmFT5a*) under LD conditions, thereby delaying flowering [24, 26]. This repression is promoted by *PHYA2* and *PHYA3*, which enhance *E1* expression and stabilize its protein [21]. Soybean also carries two additional *E1* homologs, *E1La* and *E1Lb*, that contribute to flowering regulation in certain genetic backgrounds [27]. However, functional divergence has been observed in other legumes: in *Medicago*, the *E1* homolog *MtE1L* activates *MtFTa1* and plays a relatively minor role in flowering time regulation [24], while in *Lotus japonicus*, natural variation in *LjE1* is associated with latitudinal flowering differences [28]. These findings suggest that, although *E1* is a conserved regulator, its role has diversified across the legume family to accommodate species-specific photoperiodic strategies.

Despite the established role of the *PHYA–E1–FT* regulatory module in soybean, the function of *E1* in common bean remains uncharacterized. To date, only *PvPHYA3* and *PvCOL2* have been identified as key genetic factors contributing to photoperiodic flowering adaptation in this species [20, 25]. Given the central role of the *PHYA–E1–FT* pathway in soybean, and the inverse function of *COL* genes in legumes compared to Arabidopsis, the potential interactions among *PvPHYA3*, *PvE1*, and *PvCOL2* warrant investigation. Understanding whether *PvE1* functions in a manner analogous to soybean *E1*, or has diverged like *E1L* in *Medicago*, could provide valuable insights into the molecular basis of photoperiodic control and legume adaptation to different environments.

The main objective of this study was to further characterize and advance our understanding of the genetic basis of photoperiod insensitivity that has enabled the adaptation of common bean to diverse latitudinal environments. Previous work identified two adjacent QTLs for flowering time on chromosome 09, which together explain over 30% of phenotypic variance [29]. Here, we combined fine-mapping, expression profiling, and the use of near-isogenic lines (NILs) to identify *PvE1* as a strong candidate regulator of flowering time in common bean. Our results indicate that *PvE1* is associated with delayed flowering under LD conditions through repression of *PvFTa3*, *PvFTb1*, and *PvFTc*. Collectively, our findings provide new insights into the genetic control of photoperiod sensitivity and position *PvE1* as a promising genetic target for breeding early-flowering, day-neutral cultivars adapted to temperate and high-latitude agroecosystems.

## Results

### Fine-mapping of flowering time QTLs identifies candidate gene on chromosome 9

In a previous study, two major QTLs associated with flowering time were mapped to chromosomes 4 and 9 in a recombinant inbred line (RIL) population (BN population), which was evaluated under six LD and six SD environments [29]. The parental genotypes of BN population represented contrasting phenotypes: PHA0595 is an early-flowering, photoperiod-insensitive cultivar from Spain, whereas PHA1037 is a late-flowering, photoperiod-sensitive landrace from Bolivia. The QTL on chromosome 4 was associated with allelic variation in the *PvCOL2* gene, previously shown to influence photoperiod sensitivity in common bean [20]. On chromosome 9, two overlapping QTLs—*DTF9.4* and *DTF9.5*—were mapped, collectively explaining up to 32% of the phenotypic variation in flowering time, highlighting this region as a promising target for candidate gene discovery. To refine this interval, residual heterozygous lines (RHLs) were selected from the BN population, and near-isogenic lines (NILs) were subsequently developed through marker-assisted selection (MAS), as described below.

A 223-kb candidate interval (30.83–31.06 Mb) encompassing both QTLs was delineated, containing 15 annotated genes (from *Phvul.009G203300* to *Phvul.009G204700*) according to the *Phaseolus vulgaris* v2.1 reference genome (https://phytozome-next.jgi.doe.gov/info/Pvulgaris_v2_1; Fig. S1). Sequencing of these genes in both parental lines identified polymorphisms in five genes (Tables S1–S3), which were subsequently evaluated as potential candidates. Three genes, *Phvul.009G204500*, *Phvul.009G204600*, and *Phvul.009G204700*, showed polymorphisms unique to PHA0595, relative to both PHA1037 and the reference accession G19833. *Phvul.009G204500*, encoding a fucosyltransferase, contained a SNP in exon 2, resulting in a nonsynonymous substitution of arginine (R) to lysine (K) at amino acid position 132 of the protein. However, its ortholog in Arabidopsis (*RRT1*, *AT5G15740*) is involved in seed coat mucilage development, and its relevance to flowering time remains unclear. *Phvul.009G204700*, encoding a hypothetical protein, harbors a SNP in intron 9 specific to the early cultivar PHA0595. Among these candidates, a particularly strong candidate gene within the region is *Phvul.009G204600*, which shows homology to the *E1* gene in soybean (*Glyma06g23026*) a major regulator of flowering time and photoperiod sensitivity [27, 30, 31]. This gene, henceforth referred to as *PvE1*, belongs to a legume-specific family related to the RAV subfamily of B3 transcription factors, including the Arabidopsis *TEMPRANILLO* genes, which are known repressors of *FT* expression [32]. In PHA0595, polymorphisms were identified in the 3′ untranslated region (3′-UTR) of *PvE1*, including a trinucleotide substitution (AAT to GTC), a 34-bp deletion (hereafter referred to as the *e1-del* allele) and a dinucleotide substitution (TG to GT). These polymorphisms were absent in both PHA1037 and G19833. While the coding region of *PvE1* is conserved, 3′-UTRs are known to influence mRNA stability, translation efficiency, and subcellular localization, and thus can potentially alter gene expression and function [33–35]. Given its position within the refined QTL interval, its homology to soybean *E1*—a well-characterized floral repressor—and the presence of the *e1-del* allele in the photoperiod-insensitive parent PHA0595, *Phvul.009G204600* (*PvE1*) was therefore prioritized as a strong candidate gene.

In addition, two genes located toward the proximal end of the interval, *Phvul.009G203300* and *Phvul.009G203400*, carried polymorphisms specific to the late-flowering parent PHA1037. *Phvul.009G203300,* encoding a UDP-arabinopyranose mutase 3, carried a 3′ UTR SNP in PHA1037, whereas *Phvul.009G203400*, a MADS-box transcription factor (AGL8), displayed an intronic SNP. These polymorphisms were absent in the early-flowering parent PHA0595 and in G19833. Both accessions carry the *col2* mutant allele and are able to flower under LD conditions; however, the reference accession G19833 flowers substantially later than PHA0595 under LD (91 vs. 37 days), displaying an intermediate phenotype between the two parental lines. Importantly, G19833 carries the wild-type *PvE1* allele and does not harbor the *e1-del* mutation, consistent with its later flowering under LD conditions compared to PHA0595.

A complete overview of all 15 annotated genes within the 223 kb interval is provided in Table S3, summarizing gene size, sequenced coverage, polymorphisms, expression profiles, and putative functional relevance to flowering time. Of the five genes with detected polymorphisms, only *PvE1* combines a functional connection to flowering and a mutation in the early-flowering parent, further supporting its prioritization as a candidate gene for the QTL.

To validate candidate genes within the *DTF9* interval, markers were developed for the five polymorphic genes and used to genotype the BN RIL population of 185 F_2:7_ lines. From this, recombinant heterozygous lines (RHLs) were identified, including lines with contrasting *PvCOL2* alleles to assess potential epistatic interactions. *PvCOL2* underlies the major photoperiod QTL detected on chromosome 4 in the BN population and has a strong effect on flowering time. Given this strong effect, it was therefore hypothesized that *PvE1*'s influence might be partially masked in the presence of a functional *PvCOL2* allele.

Two RHL groups were selected: one with the *col2* mutant background (RHLs 005, 017, 026, 102, 137, and 184; Fig. S2A) and one with the *COL2* wild-type background (RHLs 028 and 076; Fig. S2B). Under LD conditions, most *col2* background RHLs displayed narrow segregation for flowering time (22–29 days), consistent with the early-flowering parent PHA0595. However, RHL026 was a notable exception, showing a broad segregation range of 58 days (Fig. S3). In the *COL2* background, RHL076 exhibited an even broader segregation range of 82 days, more than double that of the late-flowering parent PHA1037 (DTF = 39 days).

Both RHL026 and RHL076 harbored heterozygous segments across the *DTF9* region and were otherwise homozygous genome-wide, as confirmed by genotyping with 278 polymorphic markers distributed across the 11 chromosomes. One heterozygous plant from each RHL was selfed to generate segregating progeny, which were genotyped using markers at the five candidate genes and phenotyped for flowering time. Seed-increase generations were then combined with iterative MAS, selecting in each cycle plants that remained heterozygous at *PvE1* while progressively fixing the flanking loci. After four selection cycles for RHL026 and two for RHL076, this strategy yielded ‘base NILs’ segregating only at *PvE*1. Subsequent selfing of these ‘base NILs’ followed by progeny genotyping produced six contrasting NILs: NIL026-*e1*, NIL026-*E1*, NIL026-*het*, and NIL076-*e1*, NIL076-*E1*, NIL076-*het* (Fig. S4, Table S4).

### Comparison between NILs reveals that *PvE1* delays flowering and influences plant architecture

**Figure 1:**
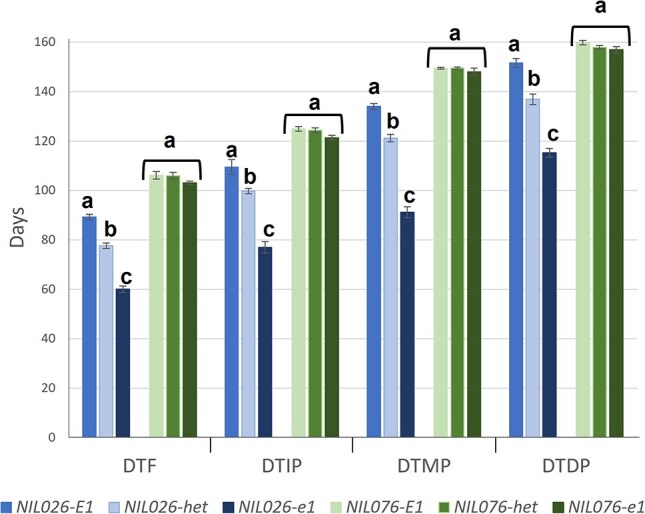
Interaction between *PvE1* and *PvCOL2* alleles in the regulation of flowering time and maturity-related traits time in near-isogenic lines (NILs). Phenotypic comparisons of NILs derived from RHL026 (*col2* mutant background; *NIL026*, shown in blue) and RHL076 (*COL2* wild-type background; *NIL076*, shown in green) grown under natural long-day conditions (LD2020, 15 h light; Pontevedra, Spain; see Table S5). In the *col2* background (RHL026), NILs homozygous for the *e1* allele flowered 29 days earlier than NILs carrying the wild-type *E1* allele, with heterozygous NILs (*NIL026-het*) showing intermediate phenotypes. In contrast, in the *COL2* background (RHL076), NILs carrying *e1*, *E1*, or heterozygous alleles did not differ significantly in flowering time or maturity traits, indicating an epistatic masking effect of *PvE1* by *PvCOL2*. Each bar represents the mean ± standard error (SE). Statistically significant differences (*P* ≤ 0.05) were determined using one-way ANOVA followed by Tukey’s HSD test; different lowercase letters indicate statistically distinct groups. Abbreviations: DTF, days to flowering; DTIP, days to immature pod; DTMP, days to mature pod; DTDP, days to dry pod.

In the *col2* background (RHL026), NILs homozygous for the *e1-del* allele flowered, on average, 29 days earlier than NILs homozygous for the *E1* allele under natural LD conditions (LD2020, 15 h; Tables S5 and S6, Fig. 1). They also matured earlier, with *NIL026-het* plants showing intermediate phenotypes—consistent with a dose-dependent loss-of-function model for *PvE1*. Conversely, in the *COL2* wild-type background (RHL076), no significant differences in flowering or maturity time were observed across *e1*, *E1*, or heterozygous NILs. This lack of phenotypic segregation suggests that *PvE1*’s effect may be epistatically masked by *PvCOL2*, as shown by the non-additive effect on flowering time. These findings support the candidacy of *PvE1* as a photoperiod response gene in common bean and indicate an epistatic relationship between *PvCOL2* and *PvE1*, in which a functional *PvCOL2* allele appears to diminish/attenuate the flowering repression mediated by *PvE1*.

The sensitivity to photoperiod in common bean is reflected not only in flowering time but also in plant architecture and the position of the first flowering node. To further elucidate the role of the *PvE1* gene in regulating these traits, particularly under varying photoperiods, both parental lines and their corresponding NILs were evaluated across multiple LD and SD environments (Tables S5 and S6).

**Figure 2:**
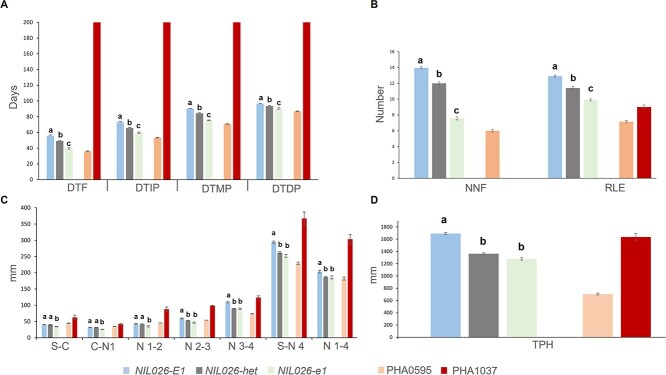
Phenotypic characterization of *E1/e1* NILs and parental lines under natural LD conditions (LD2021, 14 h light/10 h dark; Pontevedra, Spain; see Table S5). NILs derived from RHL026 (*col2* background) and parental lines (PHA0595 and PHA1037) were evaluated. **(**A) Days to flowering (DTF), immature pod (DTIP), mature pod (DTMP), and dry pod (DTDP). (B) Number of nodes to first flower (NNF) and Ratio of Leaves Expanded (RLE). (C) Internode lengths (in mm) measured for successive early stem segments (Soil–Cotyledonary node, Cotyledonary node–Node 1, Node 1–2, Node 2–3, Node 3–4) and cumulative lengths (Soil–Node 4 and Node 1–4). (D) Total Plant Height (TPH, in mm). Data are shown as mean ± standard error (SE). PHA1037 did not flower (DTF assigned a value of 200 days) and was excluded from NNF analysis. Statistical differences among NILs were evaluated by ANOVA followed by Tukey’s HSD; different lowercase letters indicate significant differences (*P* ≤ 0.05).

In the LD2021 experiment (14 h light), *NIL026-e1* flowered 16 days earlier than *NIL026-E1* (Table 1), a timing comparable to the photoperiod-insensitive parent PHA0595, and exhibited clear differences in architectural traits. *NIL026-e1* showed fewer nodes at flowering (8 vs. 14) and reached maturity 14 days earlier (Fig. 2A and B). These early developmental traits were associated with reduced vegetative growth, including both fewer total nodes and shorter total plant height (TPH) (Fig. 2B–D). Consistent with these architectural differences, *NIL026-E1* plants also exhibited a higher ratio of leaves expanded (RLE), reflecting a more advanced developmental stage at flowering (Fig. 2B). These results suggest that *PvE1* contributes to normal vegetative development. NIL comparisons revealed an average TPH difference of 24.5% between isolines: *NIL026-E1* plants averaged 1692 mm, while *NIL026-e1* plants averaged 1278 mm, with *NIL026-het* showing an intermediate phenotype (1362 mm), indicating partial dominance of the *e1-del* allele (Fig. 2D). The shorter stature of *NIL026-e1* was attributed to both fewer nodes along the main stem and reduced internode lengths (Fig. 2C), highlighting the dual role of *PvE1* in regulating flowering and vegetative development. Across LD conditions, NILs carrying the *e1-del* allele exhibited an average flowering acceleration of 29%, a 46% reduction in nodes of first flower, and a 25% decrease in TPH compared to *NIL026-E1* (Table 1).

**Table 1:** Summary of mean phenotypic effects of *PvE1* alleles in the LD2021 experiment (14 h light).

**Trait**	***NIL026-E1***	***NIL026-e1***	**% Reduction**	***E1* vs. *e1***
DTF	55.8 ± 0.92	39.7 ± 0.85	−28.9	***
NNF	14.0 ± 0.14	7.6 ± 0.21	−45.7	***
TPH	1692 ± 17.7	1278 ± 19.5	−24.5	**

Mean values ± standard errors are shown for *NIL026* lines carrying the *E1* or *e1-del* allele in the *col2* genetic background All data are presented as mean values ± standard error. DTF: Days to flowering. NNF: Number of nodes to first flower. TPH: Total plant height (in mm). Percentage reduction indicates the relative change in *NIL026-e1* compared with *NIL026-E1*. Significant differences between NILs are indicated by asterisks (^*^P < 0.05; ^**^P < 0.01; ^***^P < 0.001).

**Figure 3:**
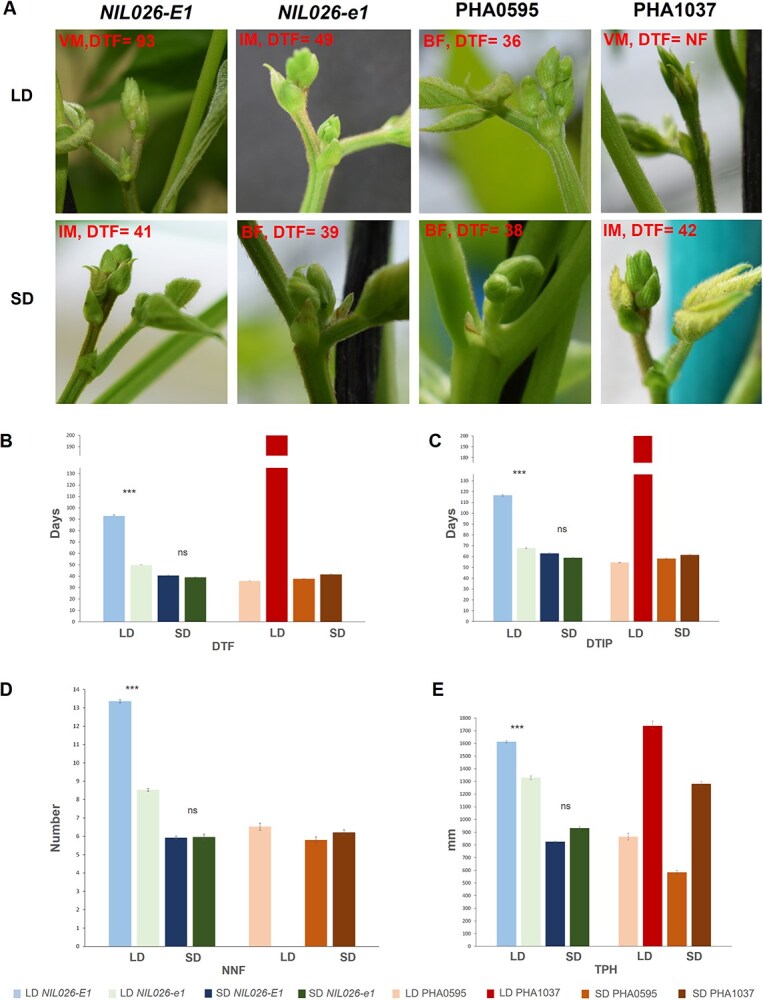
Effect of day length on flowering time and developmental traits in NILs carrying different alleles of *PvE1*. Plants were grown under long-day (LD2022, 16 h light) and short-day (SD2022, 11 h light) conditions (see Table S5). (A) Close-up images of axillary meristems at ~30 days after emergence (DAE) used as a proxy for the timing of floral induction. *NIL026-e1* and PHA0595 exhibit visible flower buds, whereas *NIL026-E1* and PHA1037 retain vegetative meristems under LD, indicating delayed floral transition. Days to Flowering (DTF) for each genotype are included in the image. VM, Vegetative Meristem; IM, Inflorescence Meristem; BF, Flower Buds. (B–E) Phenotypic analysis of DTF (B), DTIP (C), NNF (D), and TPH (E). PHA1037, which did not flower, was assigned a DTF value of 200 days. Statistical significance for *NIL026-E1* vs. *NIL026-e1* was assessed using pairwise Student’s t-test; ^***^, ^**^, and ns indicate significant differences between NILs at *P* < 0.001, *P* < 0.01, and no significant difference, respectively.

Since the effect of the *PvE1* gene seemed to increase with day length, in the following experiment, parents and both *NIL026-E1* and *NIL026-e1* were examined in a LD cycle of 16 h of light (LD2022, Table S5) and in a SD cycle of 11 h of light (SD2022, Table S5) to analyze the effect of contrasting photoperiod conditions (Fig. 3A). In the comparison between LD and SD conditions, *NIL026-E1* and the sensitive parent PHA1037 exhibited significantly delayed flowering and maturity under LD, whereas these differences were not observed under SD conditions (Fig. 3B–E). This supports a strong photoperiod-dependent effect of the *E1* allele, which is largely diminished under SD conditions. Under the 16 h LD cycle, *NIL026-e1* showed a similar pattern of developmental changes as in previous LD environments, with reduced DTF (43 days earlier), fewer nodes number (9 vs. 13) and a 17.6% of reduction in TPH compared to *NIL026*-*E1*(Table S6).

To quantify the extent to which SD conditions accelerated development, we calculated the Short-Day Hastening Rate (SDHR) for flowering and pod maturation (Table 2). The highest SDHR values were observed in the photoperiod-sensitive parent PHA1037 (79% for flowering, and 69% for pod maturation), followed by *NIL026*-*E1* (57% and 46%, respectively). In contrast, *NIL026*-*e1* showed the lowest SDHR values (21% and 12%). The photoperiod-insensitive parent PHA0595 showed no differences in flowering/pod timing between LD and SD conditions, confirming its stable developmental timing across environments.

**Table 2:** Short-Day Hastening Rate (SDHR) of common bean genotypes under varying photoperiod conditions.

**Genotype**	**SDHR_DTF %**			**LD *vs.* SD**	**SDHR_DTIP %**			**LD *vs*. SD**	**SDHR_NNF %**			**LD vs. SD**	**SDHR_TPH %**			**LD vs. SD**
NIL026-*E1*	56.6	±	0.19	***	45.9	±	0.33	***	55.4	±	0.49	***	50.6	±	0.38	***
NIL026-*e1*	20.7	±	0.57	*	12.5	±	0.65	*	31.0	±	1.01	*	34.6	±	0.65	**
PHA0595	−5.2	±	0.59		−6.4	±	0.58		7.2	±	2.02		30.9	±	2.81	**
PHA1037	79.3	±	0.26	***	69.3	±	0.23	***	NE				25.5	±	2.33	**

SDHR values (mean ± standard error) represent the percentage reduction observed under short-day (SD2022) relative to long-day (LD2022) conditions for DTF, DTIP, NNF, and TPH. For PHA1037, which did not flower by the end of the LD experiment, a DTF value of 200 days was assigned for calculations. For this genotype, NNF could not be evaluated under LD conditions due to lack of flowering (NE, not evaluated). SD and LD values were obtained from Table S5. Significant differences between LD and SD conditions are indicated by asterisks (^*^P < 0.05; ^**^P < 0.01; ^***^P < 0.001).

Together, these results reinforce the conclusion that *PvE1* gene strongly influences photoperiod sensitivity, with the *e1-del* allele promoting early flowering and reduced vegetative growth under LD conditions, while having minimal effect under SD conditions. However, because PHA0595 is less responsive to LD than *NIL026-e1* line, despite both carrying the *e1*-*del* allele, this suggests that additional photoperiod-response loci are likely mutated in PHA0595. Lastly, SD conditions caused a general reduction in plant height across all genotypes, including PHA0595, suggesting that photoperiod also independently affects vegetative growth beyond the action of *PvE1*.

### *PvE1* acts downstream of *PvPHYA3* and in parallel with *PvCOL2* to regulate florigen gene expression

We analyzed the rhythmic expression of *PvCOL2*, *PvPHYA3* and *PvE1* in the *NIL026* lines and their parental lines grown under LD conditions (16 h light/8 h dark; LD2023, Table S5). *PvCOL2* expression followed a circadian rhythm, showing three distinct peaks: one at zeitgeber time (ZT) 4 (4 h after lights on), another at ZT12, and a third early in the night at ZT20 (Fig. S5A). Although the rhythmic pattern was conserved across all genotypes, the overall expression levels of *PvCOL2* were lower in NILs and the photoperiod-insensitive parent PHA0595—both carrying the *col2* allele—compared to the photoperiod-sensitive parent PHA1037 (*COL2*). This difference was most evident at the ZT4 peak. Notably, only PHA1037 displayed significant variation in *PvCOL2* expression between LD and SD conditions across the diurnal cycle (Fig. S5B), suggesting that *PvCOL2* expression is modulated by photoperiod in a genotype-dependent manner.

*PvPHYA3* also exhibited rhythmic expression under LD conditions, with a conserved pattern and comparable expression levels among NILs and parental lines (Fig. S5C). This uniformity aligns with the shared *PvPHYA3* allele and haplotype present in all genotypes, which lack the loss-of-function mutation previously reported in both parental lines [20]. *PvPHYA3* expression was significantly higher under LD than SD conditions across all genotypes (Fig. S5D), in agreement with its established role in promoting photoperiod-dependent repression of flowering in common bean [20, 25]. The similarity in *PvPHYA3* and *PvCOL2* expression rhythms across NILs suggests that additional factors, such as *PvE1*, are likely responsible for the observed differences in flowering time and photoperiod sensitivity.

**Figure 4:**
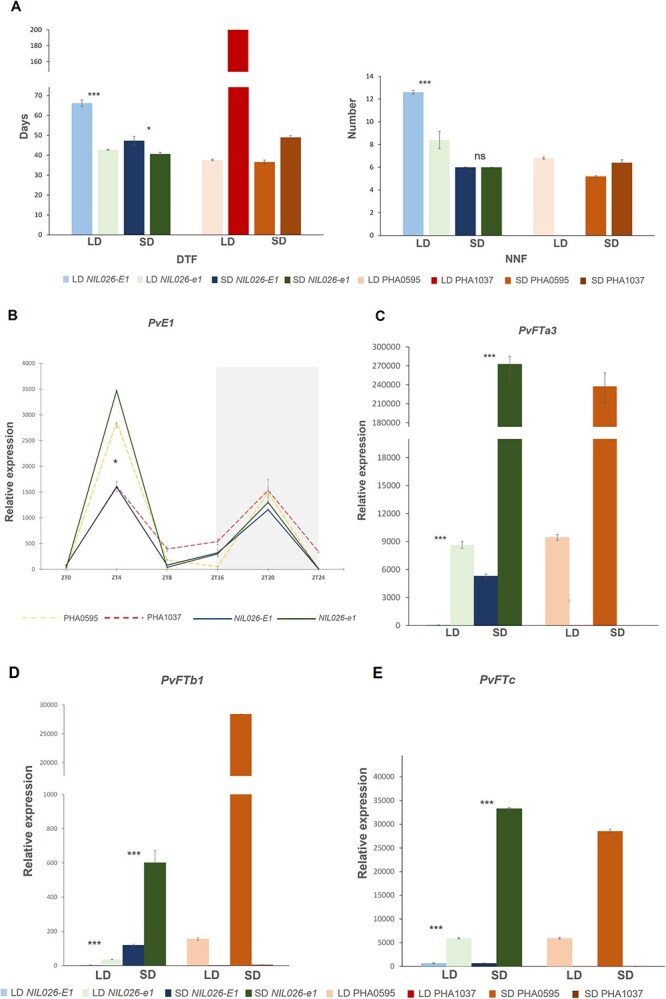
*PvE1*-dependent regulation of flowering time and *PvFT* gene expression under LD and SD conditions. (A) Days to flowering (DTF) and number of nodes to first flower (NNF) for *NIL026* and parental lines grown under LD (16 h light) and SD (11 h light) conditions (LD2022/SD2022; Table S5). PHA1037 did not flower under LD (DTF = 200), and NNF was not determined. (B) Diurnal expression profile of *PvE1* under LD (ZT0–ZT24). Only the ZT4 time point showed significant differences between NILs. Gray background indicates dark period. (C–E) Relative expression levels of *PvFTa3*, *PvFTb1*, and *PvFTc* under LD and SD conditions. Expression was quantified by RT–qPCR and normalized to UBIQUITIN. Bars represent mean ± SE of three biological replicates (each measured in three technical replicates). Samples for (C–E) were collected at ZT4 under both photoperiods. Significance between NILs within each photoperiod is indicated as ^***^, ^**^, ^*^, or ns.

Consistent with earlier greenhouse findings, *NIL*026-*E1* and PHA1037 exhibited strong photoperiod sensitivity when grown under controlled indoor LD and SD conditions (LD2022 and SD2022). *NIL026*-*E1* flowered at 66 days under LD and 47 days under SD, while PHA1037 failed to flower under LD (NF) and flowered at 49 days under SD (Fig. 4A; Table S6). In contrast, *NIL026-e1* and PHA0595, both photoperiod-insensitive, showed minimal differences across photoperiods, flowering at 42 and 43 days (*NIL026*-*e1*) and 37 and 38 days (PHA0595) under LD and SD, respectively. The 24- and 4-day earlier flowering of *NIL026-e1* compared to *NIL026-E1* under LD and SD, respectively, confirms that the *e1-del* allele reduces photoperiod sensitivity, even under controlled artificial photoperiods. Supporting this, the number of nodes to first flower (NNF) differed more between LD and SD in *NIL026-E1* (13 vs*.* 6 nodes) than in *NIL026-e1* (8 vs*.* 6 nodes), highlighting a stronger photoperiodic influence on floral initiation associated with the *E1* allele.

*PvE1* expression under LD conditions displayed a characteristic bimodal pattern with peaks in the early morning (ZT4) and early night, resembling expression profiles reported for *E1* in soybean [26, 27]. Transcript levels declined throughout the dark phase, reaching their lowest point before dawn (Fig. 4B). A significant difference between NILs was detected only at ZT4 (*P* = 0.023), when the photoperiod-insensitive *NIL026-e1* showed higher *PvE1* transcript levels than *NIL026-E1*; the parental lines PHA0595 and PHA1037 showed a similar trend. This apparently ‘paradoxical’ increase in transcript abundance in genotypes carrying the *e1-del* mutation is consistent with the self-repression model described for soybean *E1* homologues, in which loss or weakening of reduced repressor activity disrupts negative feedback. As a result, reduced autorepression of *E1* and its homologues can lead to higher transcript levels, consistent with observations that transcripts of *E1* family genes are elevated in *e1* backgrounds relative to functional *E1* alleles [36]. No alternative splicing events were detected for *PvE1* in the NILs or parental lines under the conditions tested, suggesting that the *e1-del* mutation, although it does not alter the *PvE1* coding sequence, compromises *PvE1* function through the 3′UTR deletion.

To investigate whether other genes within the *DTF9.4/DTF9.5* interval could contribute to the QTL effect, we quantified at ZT4 the expression of the remaining 14 annotated genes in this region in *NIL026* and parental lines under LD and SD (Fig. S6). Eight genes (*Phv09203300*, *Phv09203600*, *Phv09203700*, *Phv09204000*, *Phv09204100*, *Phv09204200*, *Phv09204500*, and *Phv09204700*) showed no significant expression differences between NILs under either photoperiod. Three genes (*Phv09203500*, *Phv09204300* and *Phv09204400*) displayed significant differences between NILs under LD, but these effects were not consistent with the parental lines, which showed similar expression in both environments. Two genes (*Phv09203800* and *Phv09203900*) also differed between NILs, but with opposite allelic effects when comparing NILs and parental lines (e.g., higher expression in the late *NIL026-E1* together with higher expression in the early parent PHA0595). Finally, *Phv09203400* displayed significant differences between NILs, but these were minor compared with a pronounced expression peak observed exclusively in the early parent PHA0595 under SD, whereas the late parent PHA1037 and both NILs displayed similarly low transcript levels under both photoperiods. Given that this gene encodes a MADS-box factor mainly implicated in floral and fruit development (Table S3) rather than photoperiod perception, this strong, genotype-specific induction in PHA0595 is more likely to reflect background-specific regulation under favorable light conditions than a consistent effect associated with the *DTF9* QTL. Importantly, the NILs do not segregate for the *Phv09203400* gene, as both carry the PHA1037 allele, excluding this locus as a causal factor of the QTL effect. Overall, these inconsistent expression patterns argue against any of these 14 genes underlaying the observed QTL effect and further support *PvE1* as the most likely causal gene for photoperiod sensitivity.

To investigate the downstream effects of *PvE1*, we analyzed the expression of the five known *FT* homologs in common bean—*PvFTa1*, *PvFTa3*, *PvFTb1*, *PvFTb3*, and *PvFTc*—which correspond to soybean homologs (*GmFT3*, *GmFT2a*, *GmFT6*, *GmFT1b*, and *GmFT5a*; [13, 18, 25]). Expression analysis revealed that *PvFTa3*, *PvFTb1*, and *PvFTc* were significantly more highly expressed in genotypes harboring the *e1-del* allele compared with those carrying the *E1* allele under both LD and SD conditions (Fig. 4C–E). These results are in line with observations in soybean, where the homologs *GmFT2a* and *GmFT5a* act as floral activators and promote flowering under inductive SD conditions [13, 37]. As in soybean, these common bean *FT* transcripts were generally more abundant under SD than LD.

Interestingly, while *PvFTb1* shares structural similarity with *GmFT6*, which acts as a floral repressor in soybean [38], its expression in common bean aligns more with that of an inducer. This divergence suggests a possible functional shift of *FT* homologs between species. These results highlight a potential functional divergence of *PvFTb1* in common bean, diverging from its presumed repressive role in soybean, and may indicate the need for species-specific functional validation of *FT* homologs. In contrast, *PvFTa1* showed no significant expression differences between NILs, though PHA0595 displayed elevated expression under SD, suggesting the involvement of additional genetic regulators in this background (Fig. S7A). *PvFTb3* expression was similar between NILs under LD, but higher expression was observed in *NIL026*-*E1* under SD (Fig. S7B). Across all genotypes, *PvFTb3* was more strongly expressed in LD than SD, with the highest levels in the photoperiod-sensitive PHA1037. This pattern mirrors that of the soybean homolog *GmFT1b*, a known floral repressor [13], suggesting that *PvFTb3* in common bean may also function as a floral repressor.

Together, these results indicate that *PvE1* modulates flowering, at least in part, independently of *PvCOL2* by repressing key *PvFT* genes, and suggest that functional divergence among *FT* homologs may contribute to differences in flowering behavior across legume species.

**Figure 5:**
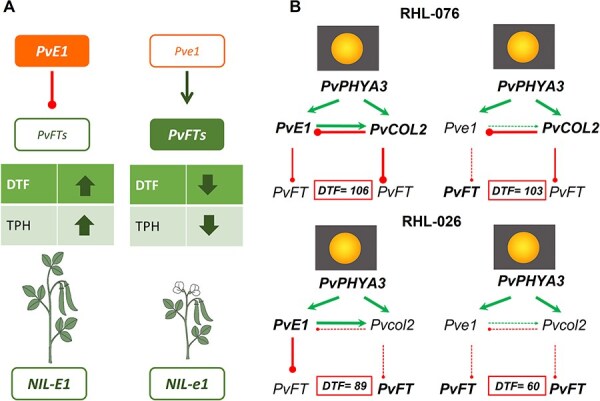
Proposed regulatory model of *PvE1* and *PvCOL2* mediated control of flowering time under long-day (LD) conditions in common bean. (A) Schematic summary of the phenotypic effects of functional and non-functional *PvE*1 alleles on days to flowering (DTF) and total plant height (TPH). In *NIL026-E1*, functional *PvE1* represses *PvFT* genes (left box unfilled), resulting in delayed flowering (↑DTF) and increased plant height (↑TPH). In contrast, in *NIL026-e1 PvFT* genes are expressed (right box filled/shaded (green)), leading to early flowering (↓DTF) and reduced height (↓TPH). (B) Gene regulatory model illustrating the independent but converging roles of *PvCOL2* and *PvE1* in repressing *PvFT* gene expression under LD conditions. Each pathway diagram corresponds to a different RHL genotype (LD2020, 15 h; see Table S5), representing combinations of functional and non-functional alleles (*E1/e1, COL2/col2*) and their corresponding flowering time (DTF, in days). *PvPHYA3,* activated by light (sun icon), promotes the expression of both *PvCOL2* and *PvE1* (activating arrows). These two regulators act in parallel to repress the expression of *PvFT* genes (non-bold), delaying flowering (blunt-ended inhibitory lines). The thickness of the arrows or lines indicates the functional status of the gene: thick lines correspond to active regulation by a functional gene, while thinner lines indicate reduced or absent regulatory influence due to gene dysfunction or repression by another gene, resulting in lower gene expression. The combined loss of function in both *PvCOL2* and *PvE1* (RHL026-*e1/col2*) results in the earliest flowering phenotype by activation of *PvFT* genes (bold), supporting the model of parallel and additive repression pathways converging on *PvFT* regulation.

Based on these expression patterns, *PvCOL2* and *PvE1* appear to act in parallel pathways to converge on the repression of *PvFT* gene expression (Fig. 5). This regulatory configuration is consistent with observations in soybean where *GmCOL1a*/*GmCOL1b* and *GmE1* function in a similar way. Indeed, overexpression of *GmCOL1a* suppresses *GmE1* expression, while *GmE1*, in turn, promotes the transcription of *GmCOL1a* and *GmCOL1b*, supporting a reciprocal regulatory relationship between these genes [17].

### Genetic variants in the *PvE1* gene and their association with flowering time in common bean

To investigate whether the deletion identified in the *PvE1* gene (designated as *e1-del*) is genetic pool-specific and to detect additional mutations affecting this locus potentially present in early-flowering accessions, we sequenced the *PvE1* gene in a diverse panel of 139 cultivars and domesticated accessions carrying nonfunctional *PvCOL2* alleles (Table S7). Among these accessions, 51 displayed a sequence identical to the wild-type *E1* allele (Haplotype 1, Table S8), while 83 accessions carried two SNPs located in the 5′ and 3′ flanking regions (Haplotype 2). In addition to the previously identified Spanish cultivar PHA0595, we identified a second Spanish cultivar, one Portuguese accession, and one breeding line from Colombia that shared the same *e1-del* allele (Haplotype 3, Tables S7 and S8). These four accessions all belonged to the Andean gene pool of common bean. We also identified a fourth *E1* variant, designated *e1-fs* (Haplotype 4), characterized by a 4-bp deletion within the coding region that introduces a frameshift at codon 127, resulting in a premature stop codon (yielding a truncated protein of 142 aa compared to the 173 aa in the wild-type *E1*). The *e1-fs* haplotype was found only in a single landrace from Mexico, belonging to the Mesoamerican gene pool.

To quantify photoperiodic effects in this germplasm panel, we analyzed flowering time under LD and SD conditions (LD2022 and SD2022), as well as growth habit. In addition, two specific photoperiod response traits were evaluated: the Photoperiod Response Index (PRI), and the Photoperiod Response Classification (PRC). Together, these traits provided a comprehensive assessment of variation in flowering behavior. Analysis of variance revealed significant differences among haplotypes for DTF under LD conditions (*p = 0.0017*), PRI (*p < 0.0001*), and PRC (*p < 0.0001*). In contrast, no significant differences in flowering time were detected under SD conditions, where all accessions flowered within 70 days. Lastly, as the groups encompassed a range of growth habits, no significant differences in growth form were observed between them. Both *e1-del* and *e1-fs* genotypes exhibited a similar flowering phenotype: all five accessions flowered earlier under LD than under SD conditions, resulting in negative or near-zero PRI values (Table S7).

Boxplot analysis (Fig. S8) showed that accessions with haplotypes 3 and 4 consistently exhibited the lowest values for DTF under LD, PRI, and PRC. These accessions flowered earlier under LD conditions and showed little or no delay in flowering compared to their performance under SD. PRI values were close to or below zero, and PRC scores were uniformly equal to 1, indicating a complete absence of photoperiod sensitivity. In contrast, accessions belonging to haplotypes 1 and 2, which carry functional *E1* alleles, displayed a broad range of photoperiod responses, including accessions with strong delays in flowering under LD (PRI values up to 98 and PRC scores up to 7). The differences among haplotypes were confirmed by Tukey’s HSD post hoc test following ANOVA, which grouped haplotypes 3 and 4 separately from haplotypes 1 and 2 for all three traits (Table S9).

**Figure 6:**
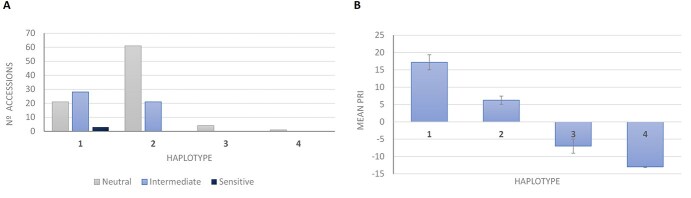
Effect of *PvE1* haplotypes on photoperiod response in common bean. (A) Frequency distribution of accessions within each *PvE1* haplotype (H1–H4) categorized by Photoperiod Response Classification (PRC): Neutral (1–2), Intermediate (3–4), and Sensitive (5–8). Haplotypes 3 and 4 (non-functional *PvE1* alleles) are exclusively classified as neutral. (B) Mean Photoperiod Response Index (PRI) per haplotype (PRI = DTF_LD – DTF_SD). Error bars represent standard deviation. Higher PRI values indicate stronger flowering delay under LD.

To further dissect haplotype-specific effects, we examined the frequency distribution of PRC categories—Neutral (1–2), Intermediate (3–4), and Sensitive (5–8)—within each haplotype group (Fig. 6A). Haplotype 1 displayed the phenotypic diversity, with 40.4% of accessions classified as neutral, 53.8% as intermediate, and 5.8% as sensitive. Haplotype 2 accessions were predominantly neutral (74.4%) and intermediate (25.6%). In contrast, all accessions assigned to haplotypes 3 and 4 were uniformly classified as neutral, reinforcing the role of *PvE1* allelic variation as a key determinant of photoperiod sensitivity.

Mean PRI values per haplotype were also calculated to assess the magnitude of the flowering delay under LD relative to SD (Fig. 6B). Haplotypes 3 and 4 showed negative or near-zero mean PRI values, while haplotypes 1 and 2 showed significantly higher means. This pattern further supports that the photoperiod response in common bean is strongly determined by allelic variation at the *PvE1* locus.

**Figure 7:**
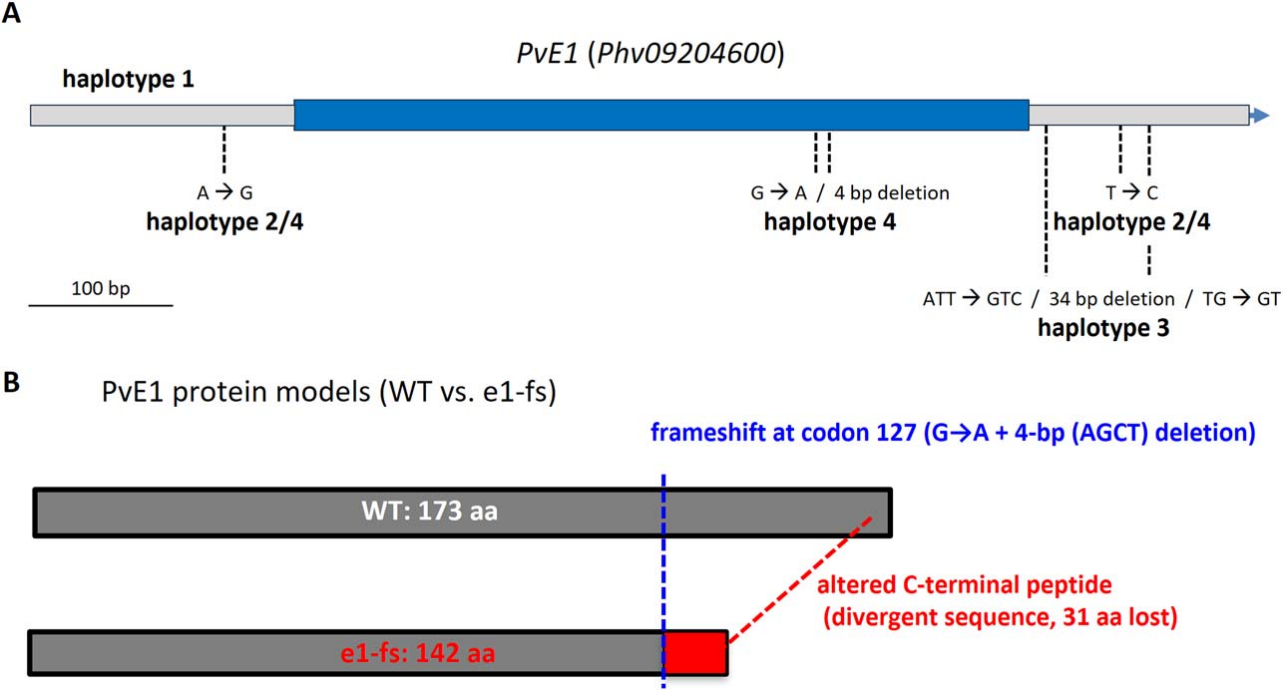
Sequence polymorphisms at *PvE1* locus. (A) Schematic representation of the *PvE1* gene, which consists of a single coding exon. Coding sequence (central segment; shown in blue) flanked by 5′ and 3′ UTRs (terminal segments; shown in grey), together with the positions of the variants defining the four *PvE1* haplotypes: Haplotype 1 (wild-type allele, PHA1037 and the reference line G12873), Haplotype 2 (SNPs in the 5′ and 3′ regions), Haplotype 3 (*e1-del*, 34-bp deletion in the 3′ UTR) and Haplotype 4 (*e1-fs*, frameshift mutation in the coding region). The corresponding SNP positions and haplotype profiles are summarized in Table S7. (B) Predicted protein products for the wild-type (WT, 173 aa) and e1-fs (142 aa) alleles. In Haplotype 4, G → A substitution together with a 4-bp AGCT deletion in the coding sequence cause a frameshift at codon 127. This frameshift replaces the conserved C-terminal region with a shorter and divergent peptide in the e1-fs protein.

The identification of two *PvE1* alleles associated with reduced photoperiod sensitivity provides further mechanistic insight into how natural variation at this locus shapes flowering responses. The *e1-fs* allele (Haplotype 4) carries a coding-region frameshift mutation that yields a truncated protein lacking part of the C-terminal region, consistent with loss of repressor function (Fig. 7; Fig. S9). In contrast, the *e1-del* mutation (Haplotype 3) affects to the 3′ UTR and is therefore unlikely to directly affect protein primary structure (amino-acid sequence). Its effect may instead arise from change in post-transcriptional regulation, such as mRNA stability, polyadenylation, translation efficiency, or subcellular localization, which could change *PvE1* protein abundance and downstream repression activity [33–35]. These potential effects are compatible with the differences in *PvE1* transcript levels observed between *e1-del* and *E1* genotypes at ZT4 under LD conditions (Fig. 4B). In soybean, functional variation at *E1* has been shown to reshape the temporal and/or quantitative dynamics of *E1* expression via changes in autoregulation [36]. This raises the possibility that allelic differences at *PvE1* could likewise influence flowering by modulating *PvE1* regulatory control, including feedback on its own expression, in common bean.

Across our germplasm panel, haplotypes 3 (*e1-del*) and 4 (*e1-fs*) are consistently associated with early flowering and photoperiod insensitivity, particularly under LD conditions (Fig. 6; Fig. S8). These alleles, which reduce or alter *PvE1* repressor activity, therefore represent valuable genetic resources for breeding early-flowering, day-neutral cultivars adapted to temperate and high-latitude environments, as well as to extended photoperiods under future climate scenarios.

## Discussion

Flowering time is a key developmental trait in common bean (*Phaseolus vulgaris*), central to adaptation, domestication, and yield stability across diverse environments. Photoperiod sensitivity plays a critical role in this process, particularly in distinguishing ancestral landraces from modern cultivars. Although multiple QTLs have been identified for flowering time, functional characterization has thus far been limited to *PvPHYA3* and *PvCOL2*, key regulators of the photoperiodic response that influence *PvFT* expression under LD conditions [20, 25]. The current study identifies *PvE1* as a novel regulator of flowering under LD, providing new insights into the molecular mechanisms underlying photoperiod sensitivity and geographical adaptation in common bean. Fine-mapping of a major QTL (*DTF9.4/DTF9.5*) on chromosome 9, identified in a cross between an early-flowering cultivar and a photoperiod-sensitive landrace, narrowed the locus to a 223-kb interval explaining ~32% of the phenotypic variation. Among the 15 annotated genes in this interval, *PvE1* emerged as the most promising candidate due to its strong sequence homology with the soybean *E1* gene, a well-characterized floral repressor. A 34-bp deletion in the 3′ untranslated region (UTR) of *PvE1* in the early-flowering parent (*e1-del* allele) was identified, which may impact mRNA stability, miRNA binding, or translation efficiency. Importantly, we further strengthened this inference by performing quantitative expression analyses at ZT4 for all annotated genes within the *DTF9* interval across NILs and parental lines under LD/SD conditions. This interval-wide survey did not support alternative candidates and instead reinforced *PvE1* as the gene whose expression pattern and allelic association best match the QTL effect. Similar regulatory effects have been reported for *E1* alleles in soybean, where noncoding variation rather than coding sequence mutations can lead to functional divergence [26, 39, 40].

Gene expression profiling showed that *PvE1* is clock-modulated under long days, displaying a clear circadian pattern with two daily expression peaks. The higher *PvE1* transcript levels observed in the *NIL026-e1* at ZT4 suggests that flowering-time differences are not simply explained by reduced *PvE1* mRNA abundance at this time, but are more consistent with reduced functional *PvE1* output. Given that *e1-del* lies in the 3′ UTR and is unlikely to alter protein primary structure, it may act through post-transcriptional mechanisms (e.g., translation efficiency, polyadenylation, or mRNA turnover). This expression pattern is compatible with the negative autoregulation reported for soybean *E1* homologues, where reduced repressor activity weakens self-repression and can lead to transcript accumulation [36]. Phenotypic characterization of NILs provided strong support for *PvE1* as a major regulator of photoperiod sensitivity, with the observed flowering responses reflecting the allelic state at *PvE1*. Plants carrying the *e1-del* allele flowered significantly earlier under LD conditions than those with the wild-type *PvE1* allele, with heterozygous individuals exhibiting intermediate flowering times, whereas no significant differences were observed under SD conditions, indicating that *PvE1’*s effect is largely LD-specific. These findings mirror results in soybean, where *E1* acts as a central hub in the flowering regulatory network, with overexpression leading to late flowering and loss-of-function mutations causing photoperiod insensitivity [26, 31]. Beyond its effect on flowering time, *PvE1* also influenced vegetative architecture. NILs carrying the *e1-del* allele were significantly shorter and had fewer nodes and shorter internodes compared to NILs carrying the wild-type allele. In soybean, *E1* overexpression increases plant height through an increase in node number, but not internode length [41], suggesting that *PvE1* may regulate distinct downstream pathways in common bean. The dual role of *PvE1* in controlling both flowering time and plant height makes it a promising target for breeding programs aiming to optimize both phenology and plant architecture.

An important finding of this study is the epistatic interaction between *PvE1* and *PvCOL2*, and their joint impact on the expression of florigen genes. In genotypes carrying a functional *PvCOL2* allele, the effect of *PvE1* on flowering time was largely masked, consistent with *PvCOL2* acting in parallel with *PvE1* in the regulatory network. The *e1col2* double-mutant genotype displayed an extremely early flowering phenotype and complete photoperiod insensitivity. This result contrasts with soybean, where *E1* function is partially redundant due to the presence of two homeologs (*E1La* and *E1Lb*), and loss-of-function mutations at *E1* alone do not fully abolish photoperiod sensitivity [27]. The absence of *E1* homologs in common bean may therefore amplify the phenotypic impact of *PvE1* mutations. From an evolutionary perspective, the conserved role of *E1* in soybean and common bean highlights its ancient function within legume flowering networks, despite differences in gene copy number and regulatory architecture between species [42]. Future research should extend to other SD legumes to evaluate the conservation and divergence of *E1-like* gene function across species.

Within this framework, the transcriptional reprogramming of *PvFT* genes provides a mechanistic link between *PvE1*/*PvCOL2* activity and flowering-time phenotypes. NILs carrying the *e1-del* allele exhibited elevated expression of *PvFTa3* and *PvFTc* under LD conditions, consistent with early flowering. Conversely, *PvE1*-expressing NILs showed strong repression of these floral inducers. These patterns mirror those in soybean, where *E1* represses *GmFT2a* and *GmFT5a* to delay flowering [13, 43]. However, divergence in *FT* homolog function was also observed: *PvFTb1* was upregulated under inductive conditions in common bean, suggesting a promotive role in flowering in contrast to the repressive function of its homolog *GmFT6* in soybean [38]. Such findings support the hypothesis that *FT* homologs in legumes have undergone neo- or sub-functionalization [44–47], resulting in an activator–repressor balance that fine-tunes the transition from vegetative to reproductive growth. Together, these results highlight the potential neofunctionalization of *PvFTb1* in common bean, diverging from its presumed repressive role in soybean, and reinforcing the need for species-specific functional validation of *FT* homologs. Our results suggest that *PvE1* operates within a gene network with *PvCOL2* and *PvPHYA3*. Expression of *PvE1* was unchanged in a *col2* mutant background, but full repression of *PvFT* genes under LD required functional alleles of both *PvE1* and *PvCOL2*. The double mutant *e1col2* displayed the highest *FT* expression and most extreme insensitivity, closely resembling *phya3* mutants [20]. Prior work showed that *PvFTa3*, *PvFTb1*, and *PvFTc* are more strongly upregulated in *phya3* than in *col2* mutants, which is consistent with *PvE1* acting downstream of *PvPHYA3* and cooperates with *PvCOL2* to suppress *PvFT* expression. These interactions resemble soybean, where *GmCOL1* expression is unaffected by *E1* alleles [48], supporting a convergent regulatory architecture.

To explore natural variation in *PvE1*, we sequenced the gene in a diverse panel of common bean accessions. In addition to the *e1-del* allele identified in the early-flowering parent used to develop our NILs, we found this same 3′ UTR variant in other early-flowering Andean accessions. We also identified a second allele, *e1-fs*, a rare Mesoamerican variant carrying a coding-region frameshift that results in premature translation termination affecting *PvE1* function. In our panel, *col2* mutations are common (~37% of Mesoamerican and ~ 90% of Andean landraces) [20], *e1* mutations are rare (~4%), possibly due to reduced selective pressure. Since *col2* alone already provides substantial photoperiod insensitivity, the advantage conferred by *e1* is partially additive but not essential. Nonetheless, accessions with both *col2* and *e1* mutations flowered ~15 days earlier than those with *col2* alone, suggesting that nonfunctional *PvE1* alleles represent untapped genetic diversity for breeding early-flowering lines in temperate or high-latitude environments. From an evolutionary perspective, this pattern suggests that alleles affecting *PvE1* function may have arisen independently and remained at low frequency due to relaxed selection in regions where *col2* already ensured adaptation to shorter growing seasons. This scenario is consistent with repeated selection toward day-neutrality in legumes cultivated at temperate latitudes. Their presence in both Andean and Mesoamerican gene pools, supports the idea that day-neutrality has been repeatedly favored under domestication and cultivation at higher latitudes. As agriculture expands into marginal environments, such alleles represent valuable, underutilized genetic resources for breeding programs aimed at accelerating flowering and securing yield stability under long-day or temperate conditions.

## Conclusions and remarks

This study provides strong evidence that *PvE1* acts as a key regulator of photoperiod sensitivity and flowering time in common bean. Fine-mapping, gene expression analyses and NIL phenotyping jointly support a model in which *PvE1* functions as a floral repressor under LD conditions, analogous to its soybean ortholog *E1*. Natural allelic variation at this locus, including the *e1-del* and *e1-fs* alleles, promotes earlier flowering—particularly when combined with nonfunctional *col2* alleles. The epistatic interaction between *PvE1* and *PvCOL2*, together with their convergent repression of *PvFT* genes, reveals a complex photoperiodic regulatory network that parallels, but is not identical to, the *E1*-based model described soybean.

*PvE1* mutations remain largely unexploited in current common bean germplasm, representing a valuable opportunity to accelerate the development of photoperiod-insensitive cultivars. Future research should extend the analysis of *E1*-like genes to other legume species and dissect the molecular mechanisms by which *E1* represses *FT* homologs, in order to clarify the evolutionary conservation and diversification of this regulatory module and to fully exploit it in legume improvement programs.

## Materials and methods

### Plant material and development of NILs

In a previous study, a RIL population of 185 F_2:7_ lines (BN population) was developed from a cross between two contrasting genotypes of common bean: PHA0595, a photoperiod-insensitive, early-flowering cultivar from Spain with a type II indeterminate growth habit and large white seeds; and PHA1037, a photoperiod-sensitive, late-flowering landrace from Bolivia with a type IV climbing growth habit and large red seeds [29]. QTL mapping in this population, conducted under both SD and LD conditions, identified two adjacent QTLs for flowering time on chromosome 9, designated as *DTF9.4* and *DTF9.5*. These QTLs span a 30-kb region and collectively explain between 2% and 32% of the phenotypic variance for flowering time across environments (Fig. S1). The target region contains 15 annotated genes.

To fine-map and validate these QTLs, two residual heterozygous lines (RHLs), RHL-026 and RHL-076, were selected from the BN RIL population based on the presence of heterozygosity within the refined *DTF9* interval and homozygosity across the remainder of the genome. Selection was performed using Sequence Characterized Amplified Region (SCAR) and derived Cleaved Amplified Polymorphic Sequence (dCAP) markers specific to the *DTF9* interval (Table S1). Marker data were visualized using GGT 2.0 software [49], and chromosomal segments representing allele distribution are shown in Fig. S2.

RHL-026 carries the *col2–1* mutation, identical to the allele found in PHA0595 [20], whereas RHL-076 retains the wild-type *COL2* allele from PHA1037. Both RHLs possess the wild-type *PHYA3* allele and are heterozygous across the *DTF9* interval. These lines displayed a broad range of flowering-time variation under LD conditions (Fig. S3).

To develop near-isogenic lines (NILs), seeds from individual heterozygous plants of RHL-026 and RHL-076 were harvested and advanced, generating the segregating progenies RHL026-05 and RHL076-10. NILs differing specifically at the *E1*/*e1* locus within otherwise fixed *COL2*/*col2* genetic backgrounds were obtained through marker-assisted recurrent selection across the for heterozygosity at *DTF9* region and parallel visual selection for phenotypic uniformity (Fig. S4). These segregating NIL populations were subsequently used for QTL validation and phenotypic evaluation.

### Phenotyping and evaluation of segregating populations and germplasm panel

Segregating populations derived from heterozygous plants retaining recombination within the DTF9 interval were used to validate and estimate the relative phenotypic effect of the major QTL *DTF9*. These trials were conducted in Pontevedra, Spain (latitude 42° 24′ 17.99″ N, longitude 8° 38′ 38.2″ W, altitude 40 m.a.s.l.) under LD and SD photoperiod conditions (see Table S5). Additionally, the photoperiod responsiveness of a panel of 139 domesticated common bean accessions (Table S7) was evaluated under controlled LD and SD conditions in a greenhouse, with two replicates per accession (Table S5). For each accession, a minimum of six individual plants were phenotyped.

The following traits were recorded for each plant: Days to Flowering (DTF), defined as the number of days from seedling emergence to the first fully developed/open flower with standard and wing petals fully unfurled; Node Number to First Flower (NNF), scored as the node bearing the first secondary inflorescence (I2) on the main stem; Days to Immature Pod (DTIP), recorded when the plants exhibited immature pods at the R4 stage (pod at maximum length, seed not yet discernible); Days to Mature Pod (DTMP), recorded as the number of days to the appearance of a pod with fully developed seeds (R6 stage); and Days to Dry Pod (DTDP), the number of days to the first dry pod at harvest maturity (R9 stage). To evaluate plant growth, Total Plant height(TPH) was measured four weeks after emergence from the base to the apex, taking the first node (nonfoliate leaf) as point zero. In addition, internodal lengths were recorded, including the distance from the soil to the cotyledonary node, from the cotyledonary node to the first node, and between successive nodes up to the fourth node. The total length from soil to the fourth node and from the first to fourth node was also measured. Finally, the Ratio of Leaves Expanded (RLE was) calculated as the number of nodes with fully expanded leaves.

The SD Hastening Rate (SDHR) was calculated to assess the effect of photoperiod on flowering time, maturity, and plant height using the following formula [50]:


$$ \mathrm{SDHR}\left(\%\right)=\frac{\mathrm{VLD}-\mathrm{VSD}}{\mathrm{VLD}}\times 100 $$

where VLD represents the phenotype value under LD conditions and VSD under SD conditions. Additionally, two further measurements were made to compare flowering time responses of the germplasm panel in both photoperiods: the PRI, quantified as VLD - VSD [51], and Photoperiod Response Classification (PRC), defined as the mean delay in flowering due to photoperiod based on a scale of 1–8 [6]. The scale is as follows: 1 ≤ 3 days delay, 2 = 4 to 10 days delay, 3 = 11 to 19 days delay, 4 = 20 to 39 days delay, 5 = 40 to 59 days delay, 6 = 60 to 79 days delay, 7 = 80 to 99 days delay, and 8 = over 100 days delay in flowering. Accessions were grouped according to PRC classes: 1 and 2 as day-neutral, 3 and 4 as intermediate, and 5–8 as sensitive. Growth habit was also recorded and categorized according to common bean classification: Type I (determinate bush), Type 2 (indeterminate upright), Type 3 (indeterminate prostrate), and Type 4 (indeterminate climbing) [52].

To evaluate the functional effect of different *E1* haplotypes identified in the panel on photoperiod sensitivity, an Analysis of Variance (ANOVA) was conducted to test for significant differences among traits. When significant effects were detected, Tukey’s Honest Significant Difference (HSD) post hoc test was applied for pairwise comparisons. Trait means, standard errors, and grouping letters based on Tukey's test were obtained using the Descriptive Statistics and ANOVA modules in XLSTAT [53]. All data visualizations, including boxplots and bar charts, were generated in Excel using the results exported from XLSTAT.

### Fine mapping of the *DTF9* locus

To verify and refine the target location of the QTL *DTF9*, intron-spanning fragments of 15 genes within the region were generated by PCR using primers designed with Primer3 (http://primer3.wi.mit.edu/ [54, 55]). These fragments were sequenced to identify suitable polymorphisms for genotyping. Full-length genes were amplified from genomic DNA in several overlapping fragments using the primers listed in Table S1. PCR products were purified and sent to Macrogen Inc. for sequencing. Sequence analysis and alignments were performed using Geneious V8.1.8 software [56] to correct miscalled bases, remove unreadable sequence at the 3′ and 5′ ends, and group sequences into contigs. Sequence identity was confirmed by BLAST searches or alignment with existing sequences.

Five new molecular markers, based on polymorphisms identified between the parental lines, were designed using dCAPs Finder 2.0 (http://helix.wustl.edu/dcaps/dcaps.html [57]) and Webcutter 2.0 (https://heimanlab.com/cut2.html) (Table S1). These markers were used for genotyping all progeny plants from the selected RHLs. One of these genes targeted was the common bean ortholog of soybean *E1*, the *PvE1* gene (*Phvul.009G204600*). NILs for *PvE1* were developed from progeny of RHL-026 and RHL-076 by MAS of *E1* heterozygotes in subsequent generations, ensuring uniformity for other genes.

### Assaying gene expression via qRT-PCR

For gene expression analysis, seeds of both NILs and their respective parents were grown in growth chambers at a constant temperature of 22°C or 25°C, under SD (8 h light/16 h dark) or LD (16 h light/8 h dark) photoperiod conditions, as detailed in Table S5. *PvE1* is primarily expressed in leaves and at low levels in other tissues [26, 58, 59]. Therefore, samples were collected from the last fully expanded trifoliate leaves of 4-week-old plants, which were immediately frozen in liquid nitrogen and stored at −80°C.

For diurnal rhythmic expression analysis, samples were taken every 4 h after lights-on (from Zeitgeber Time 0 to 24; ZT0-ZT24). *PvE1, PvCOL2*, and *PvPHYA3* expression levels were analyzed under LD conditions. The timing of the morning *FT* peak remains largely consistent across species at ZT4 [60], so plants grown under SD conditions were sampled at ZT4 for comparison. In addition, expression of all annotated genes within the *DTF9* interval and *PvFT* genes was quantified by qRT-PCR at ZT4 under both LD and SD conditions, using the same sampling and normalization procedures described above. All sampling was performed in triplicate for each time point across all samples.

Total RNA was isolated using the Trizol method (Invitrogen, China). RNA integrity was assessed electrophoretically, and RNA quality was evaluated using a NanoDrop™ ND-2000c Spectrophotometer (Thermo Scientific, Wilmington, DE, USA). Genomic DNA was removed, and complementary DNA (cDNA) was synthesized using a DNA-free™ kit (Ambion, Life Technologies, Carlsbad, CA) and the SuperScript First-Strand cDNA Synthesis System (Takara, Dalian, China).

Quantitative real-time PCR (qRT-PCR) was performed using the SYBR Green PCR Master Mix (Applied Biosystems, Foster City, CA, USA) on a 7300 Real-Time PCR System (Applied Biosystems). Reactions were carried out in duplicate with a total volume of 10 μl, containing 1 μl of cDNA and 300 nM of each primer. The thermal cycle conditions were as follows: initial denaturation at 95°C for 10 min, followed by 40 cycles of 95°C for 15 s and 60°C for 1 min. A melting curve analysis was performed at the end of each reaction to ensure the amplification of a single PCR product. Expression levels were calculated using the ∆∆Ct method [61] and were normalized to the housekeeping gene *UBIQUITIN* (*Phvul.001G193800*). Specific primer pairs used for qRT-PCR in this study are listed in Table S1.

Three independent biological replicates were conducted for each experiment, each measured in triplicate (technical replicates). Statistical significance of gene expression differences between NILs was assessed using Student’s *t*-test (ns: non-significant, ^*^ *P* ≤ 0.05, ^**^ *P* ≤ 0.01, ^***^ *P* ≤ 0.001).

## Supplementary Material

Web_Material_uhag021

## Data Availability

The data underlying this study are included in the article and its online supplementary materials.
