# Supplementary material for: PvE1 plays an essential role in regulating photoperiod sensitivity and flowering time in common bean
Source: Hortic Res. 2026 Jan 22;13(4):uhag021. doi: 10.1093/hr/uhag021 (PMC13098377; doi:10.1093/hr/uhag021)
Supplement: Web_Material_uhag021 [file web_material_uhag021.zip › Suppl.fig.R2_YellowMarkedChanges.docx]

**Supplementary Figures for:**

***PvE1* plays an essential role in regulating photoperiod sensitivity and flowering time in common bean**

Ana M. González^1, *^, Ana M. Pesqueira^1^, Rocío Fonseca^2^, Sandra Bretones ^2^, Fernando J. Yuste-Lisbona ^2^, Rafael Lozano^2^, Marta Santalla^1, *^

^1^Grupo Genética del Desarrollo de Plantas, Misión Biológica de Galicia-CSIC, PO Box 28, 36080 Pontevedra, Spain.

^2^Centro de Investigación en Biotecnología Agroalimentaria (CIAIMBITAL), Universidad de Almería, 04120 Almería, Spain.

*Corresponding authors. E-mails: [amgonzalez@mbg.csic.es](mailto:amgonzalez@mbg.csic.es); [msantalla@mbg.csic.es](mailto:msantalla@mbg.csic.es)

**Short title:** *PvE1*: regulator of photoperiod in common bean

Email address of all authors: [amgonzalez@mbg.csic.es](mailto:amgonzalez@mbg.csic.es); [ampesqueira@mbg.csic.es](mailto:ampesqueira@mbg.csic.es); [rfr770@ual.es](mailto:rfr770@ual.es); [sba557@ual.es](mailto:sba557@ual.es); [fyuste@ual.es](mailto:fyuste@ual.es); [rlozano@ual.es](mailto:rlozano@ual.es); [msantalla@mbg.csic.es](mailto:msantalla@mbg.csic.es)

**Figure S1**. **Physical map of a 223-kb candidate interval on chromosome 9, containing the QTLs *DTF9.4* and *DTF9.5*, associated with days to flowering in common bean.** The interval (30.84–31.06 Mb) includes 15 annotated genes. Genes with polymorphisms between the parental lines PHA1037 and PHA0595 are highlighted in yellow.


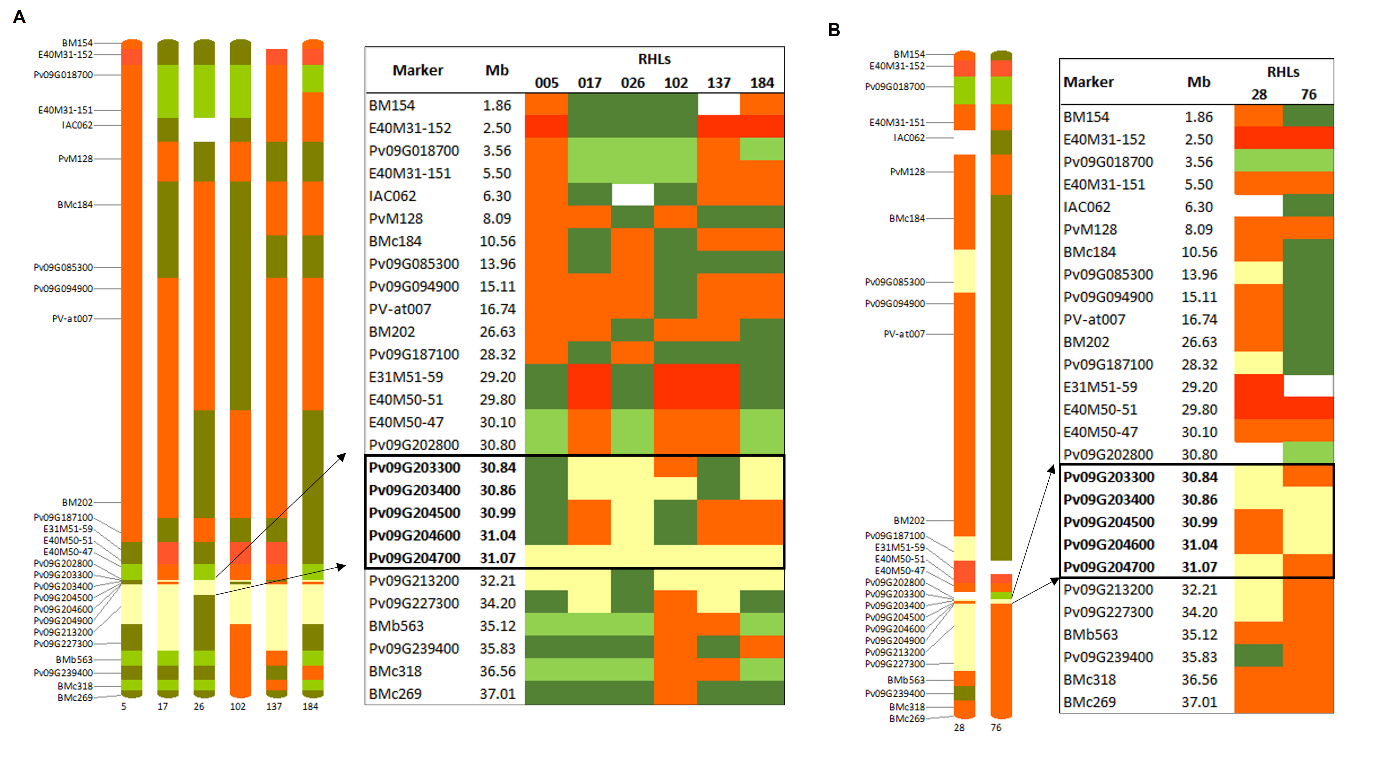


**Figure S2.** **Graphical genotypes of recombinant heterozygous lines (RHLs) across the *DTF9* region**. **(A)** RHLs with *col2* mutant background (RHLs 005, 017, 026, 102, 137, and 184). All lines except RHL026 showed narrow flowering time segregation (22–29 days) under LD conditions. **(B)** RHLs with wild-type *COL2* (RHLs 028 and 076). RHL026 and RHL076, which were heterozygous across the *DTF9* interval and homozygous at the rest of the genome, were selected for near-isogenic line (NIL) development. Marker names and physical positions (Mb) used for fine mapping are shown in bold. Five of these markers correspond to genes with polymorphisms identified within the QTL interval. Color legend: orange: codominant marker – homozygous for PHA1037 allele, dark green: codominant marker – homozygous for PHA0595 allele, yellow: codominant marker - heterozygous genotype, red: dominant marker - PHA1037 allele (in either homozygous or heterozygous state), light green: dominant marker - PHA0595 allele (in either homozygous or heterozygous state). Segregating progeny were genotyped with five candidate gene markers and phenotyped, leading to the development of six NILs: *NIL026-e1, NIL026-E1, NIL026-het, NIL076-e1, NIL076-E1*, and *NIL076-het*.


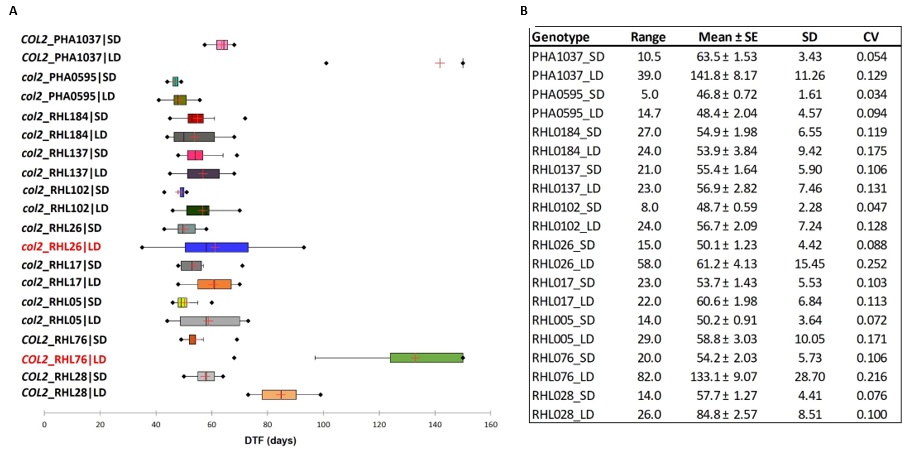


**Figure S3. Flowering time segregation in *COL2/col2* background recombinant heterozygous lines (RHLs) under long-day (LD) and short-day (SD) conditions**. (**A**) Boxplot showing phenological variation under LD and SD conditions for the two parental lines, PHA0595 and PHA1037, and selected RHLs based on molecular markers. (**B**) Mean, range, standard deviation (SD), and coefficient of variation (CV) for days to flowering (DTF) across six LD and six SD environments (data from [29]). RHLs highlighted in red correspond to RHL026 and RHL076, which exhibited the broadest flowering time variation under LD conditions and were selected for the development of near-isogenic lines (NILs).


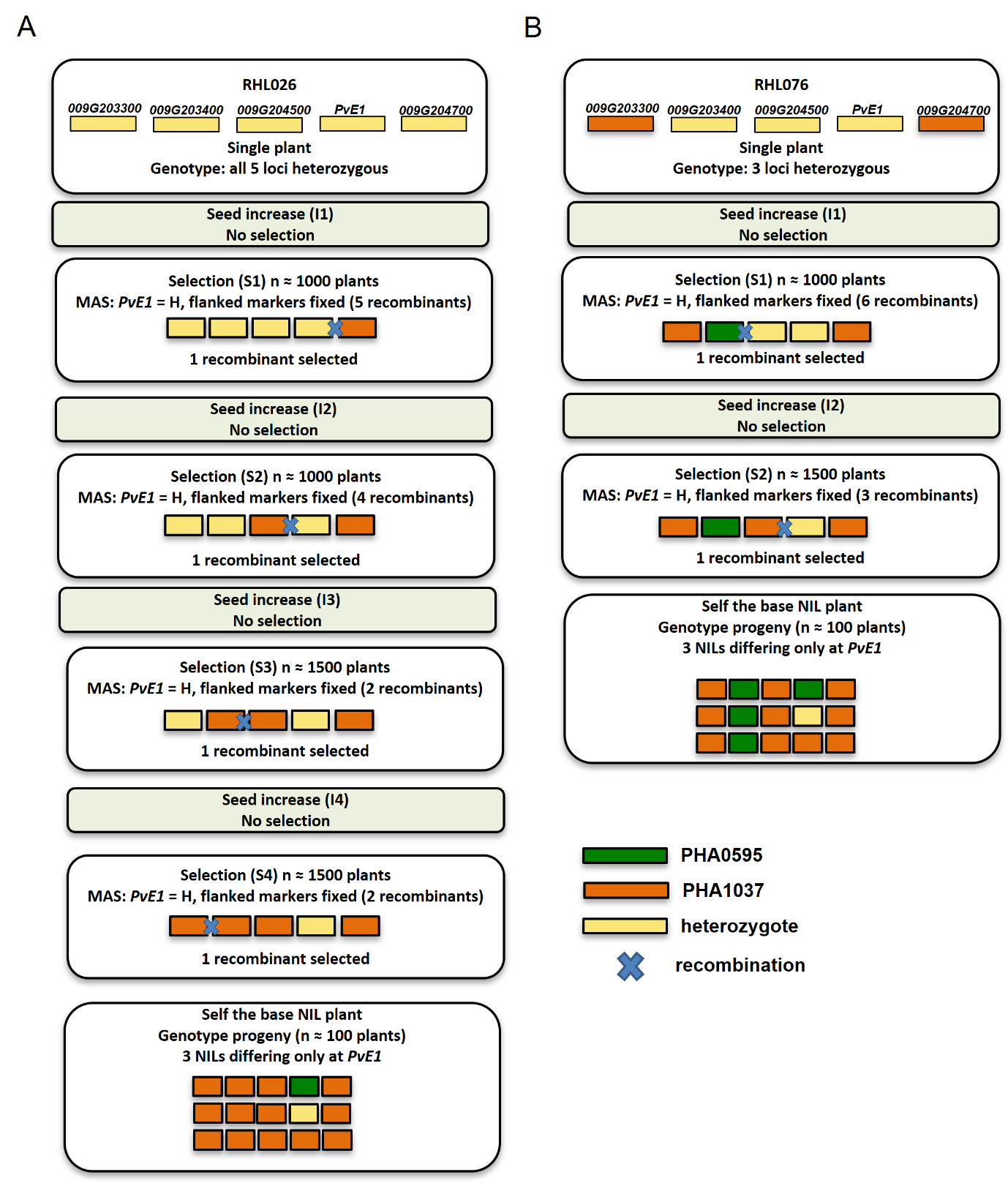


**Figure S4. Development of near-isogenic lines (NILs) targeting the *DTF9* region (30.84–31.07 Mb) on chromosome Pv09.** Two residual heterozygous lines, RHL026 and RHL076, were used to generate NILs segregating only at *PvE1* (*Phvul.009G204600*) within a five-marker interval (*Phvul.009G203300*, *Phvul.009G203400*, *Phvul.009G204500*, *PvE1* and *Phvul.009G204700*). In both cases, a single RHL plant was selfed to produce seeds, which were first bulked without selection in a seed-increase generation (I1) to obtain sufficient material for large-scale screening. In each subsequent selection cycle, up to a few thousand selfed progeny were genotyped by marker-assisted selection (MAS). MAS was applied iteratively: in each selection generation (S1–S4), plants were first identified that remained heterozygous at *PvE1* (H) and carried recombination events fixing one flanking marker; from these independent recombinants, a single plant was chosen and selfed to produce the next seed-increase generation (I2–I4). Repeating this process progressively reduced the size of the heterozygous segment until a base NIL heterozygous only at *PvE1* was obtained. Selfing the base NIL and genotyping the progeny produced three NILs differing only at *PvE1*. (**A**) Scheme for RHL026, which required four selection cycles (S1–S4). (**B**) Scheme for RHL076, which required two selection cycles (S1–S2). Orange and green boxes represent homozygous alleles for PHA1037 and PHA0595, respectively; yellow boxes indicate heterozygous loci (H); and blue crosses represent inferred recombination events.

**
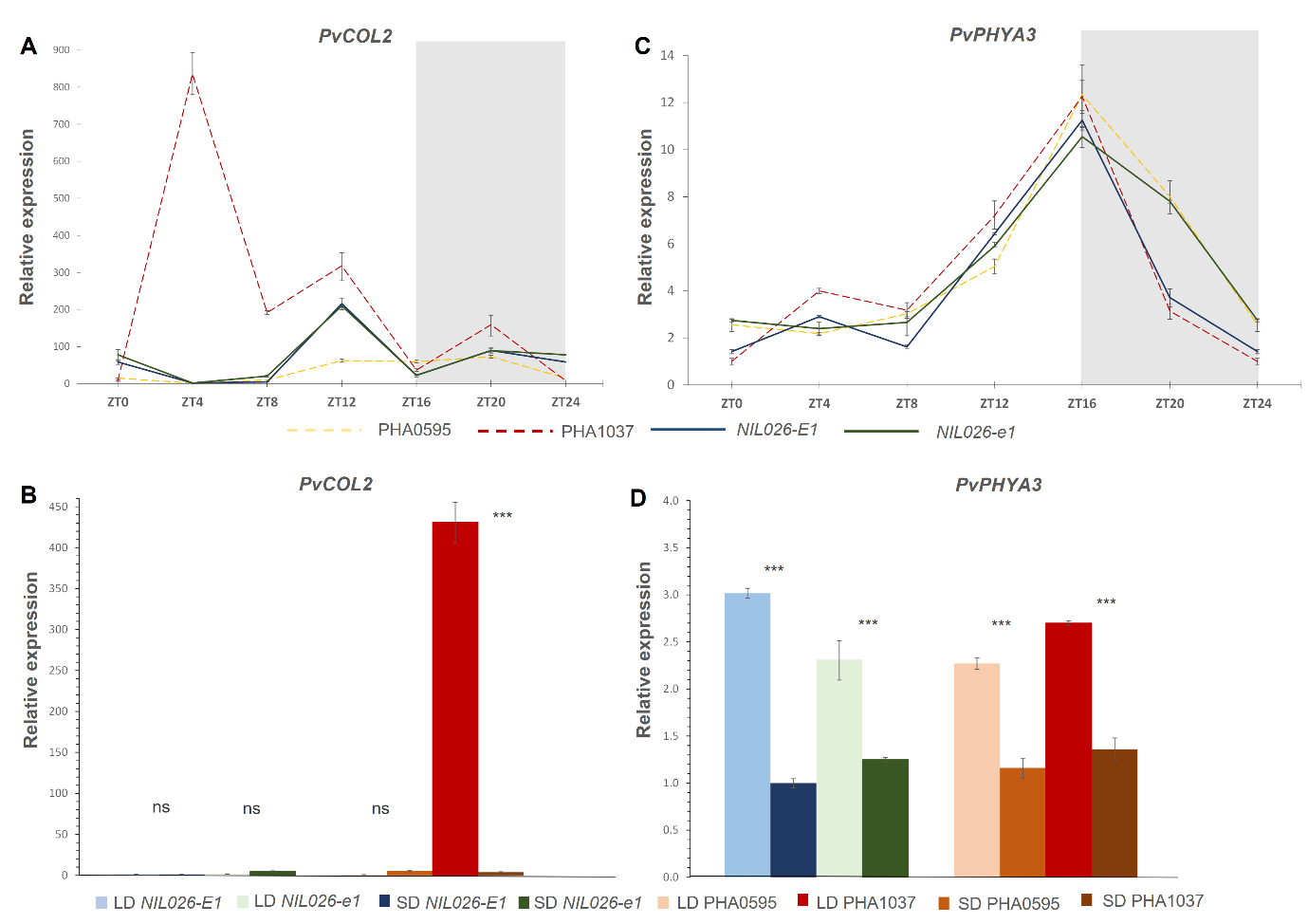
**

**Figure S5. Expression analysis of *PvCOL2* and *PvPHYA3* under contrasting photoperiod conditions. (A)** Expression pattern of *PvCOL2* under LD conditions. **(B)** Expression pattern of *PvPHYA3* under LD conditions. **(C)** Comparison of *PvCOL2* expression between LD and SD conditions. **(D)** Comparison of *PvPHYA3* expression between LD and SD conditions. Plants were grown in controlled growth chambers under either 16 h light/8 h dark (LD2023) or 8 h light/16 h dark (SD2023) photoperiods, as detailed in Supplemental Table S5. Gene expression was quantified by real-time quantitative RT-PCR and normalized to the *UBIQUITIN* housekeeping gene. Values represent the mean ± SE (n = 3) from three independent biological replicates, each comprising three technical replicates. ZT, Zeitgeber time. In panels A and B, the dark period is indicated by gray shading. ***P ≤ 0.001 (Student’s t-test); ns, not significant (panels C and D). Samples in panels C and D were collected at ZT4 (4 h after lights on) under both photoperiod conditions.


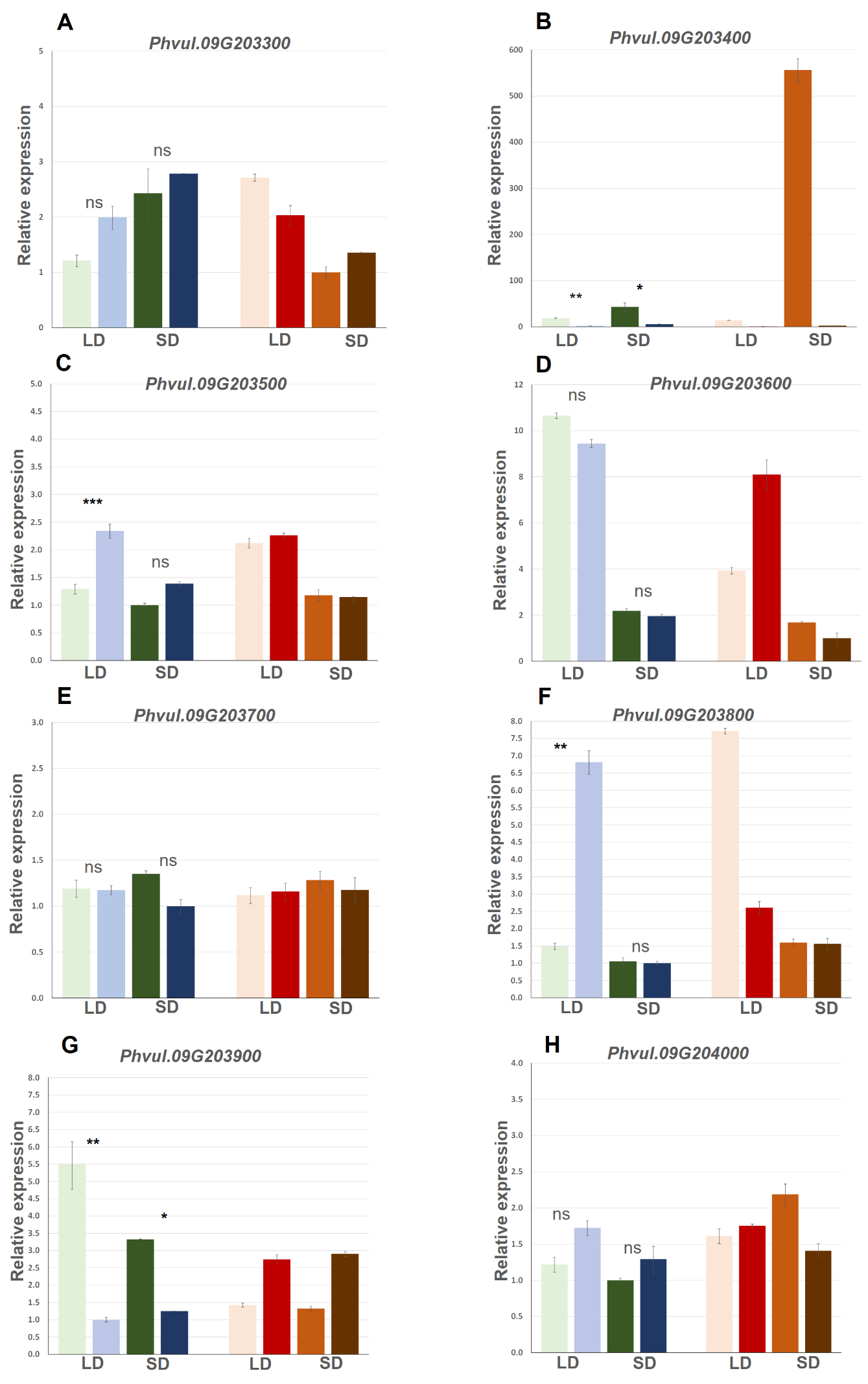


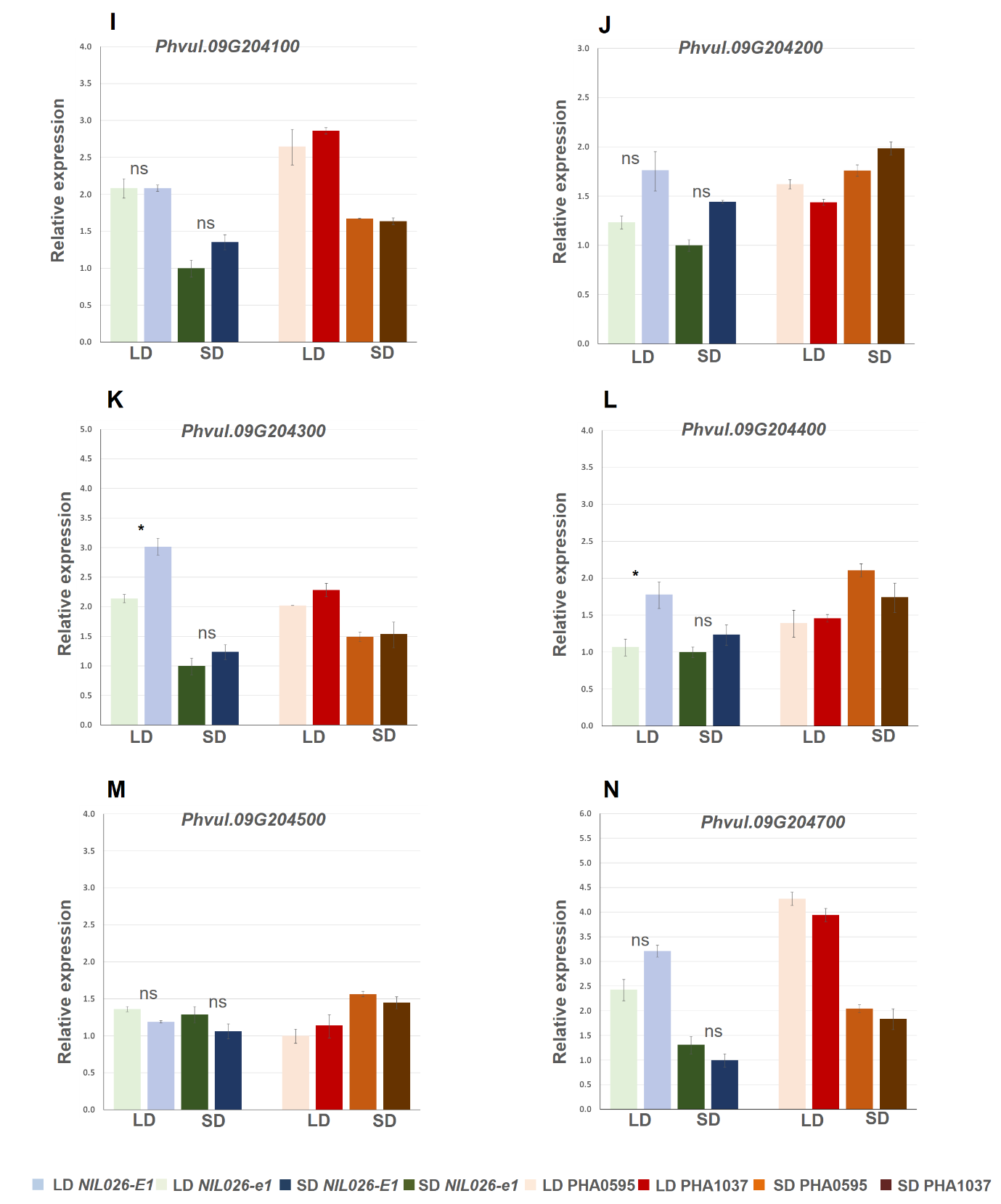


**Figure S6. Expression profiles of 14 additional genes located within the *DTF9.4/DTF9.5* interval** **under LD and SD conditions.** RT–qPCR analysis of the 14 additional genes located within the 223-kb *DTF9.4/DTF9.5* interval on Pv09 (excluding *PvE1*) in *NIL026-E1* and *NIL026-e1* and their parental lines PHA0595 and PHA1037 grown under LD (16 h light/8 h dark) and SD (8 h light/16 h dark) conditions (LD2023/SD2023; Supplemental Table S5). Samples were collected from the last fully expanded trifoliate leaf of 4-week-old plants at ZT4 (4 h after lights on). Expression was normalized as described in Materials and Methods and is presented as mean ± SE of three biological replicates. For each gene, expression patterns are shown for *NIL026-E1*, *NIL026-e1*, PHA-0595 (early parent) and PHA1037 (late parent) under LD and SD. Panels (A–N) correspond to: (**A**) *Phvul.009G203300*, (**B**) *Phvul.009G203400*, (**C**) *Phvul.009G203500*, (**D**) *Phvul.009G9203600*, (**E**) *Phvul.009G203700*, (**F**) *Phvul.009G203800*, (**G**) *Phvul.009G203900*, (**H**) *Phvul.009G204000*, (**I**) *Phvul.009G204100,* (**J**) *Phvul.009G204200*, (**K**) *Phvul.009G204300*, (**L**) *Phvul.009G204400*, (**M**) *Phvul.009G204500*, and (**N**) *Phvul.009G204700*. None of the 14 genes displayed consistent genotype-specific differences under either photoperiod. Statistical significance of expression differences between NILs was assessed using Student’s t-test (ns, not significant; P ≤ 0.05; *P ≤ 0.01; **P ≤ 0.001).


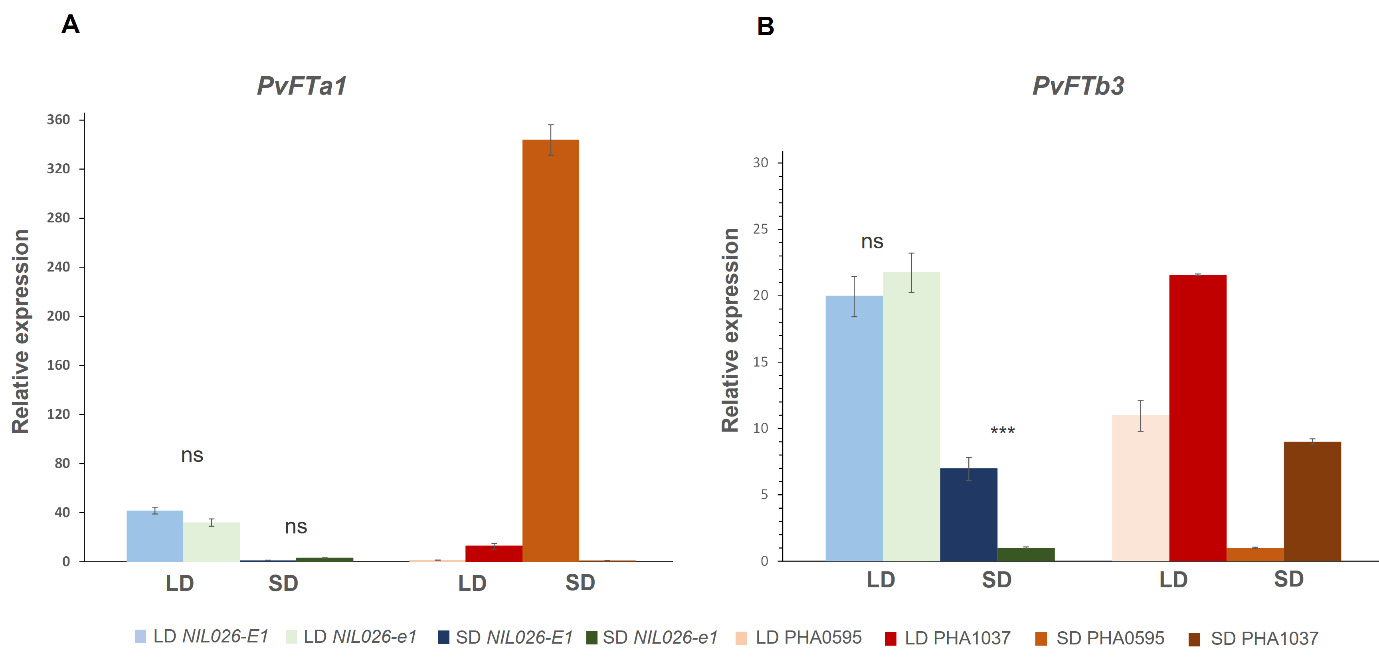


**Figure S7. Expression profiles of** **additional *PvFT*-family members under contrasting photoperiod conditions. (A)** Relative expression of *PvFTa1* under LD and SD. **(B)** Relative expression of *PvFTb3* under LD and SD. Plants were grown under LD2023 (16h light/8 h dark) or SD2023 (8 h light/16 h dark conditions (see Supplemental Table S5). Transcript levels were quantified by RT–qPCR and normalized to UBIQUITIN. Bars represent mean ± SE of three biological replicates (each measured in three technical replicates). Samples were collected at *ZT4* under both photoperiods. Significance between NILs within each photoperiod was assessed using Student’s t-test (***P < 0.001; **P < 0.01; *P < 0.05; ns, not significant).


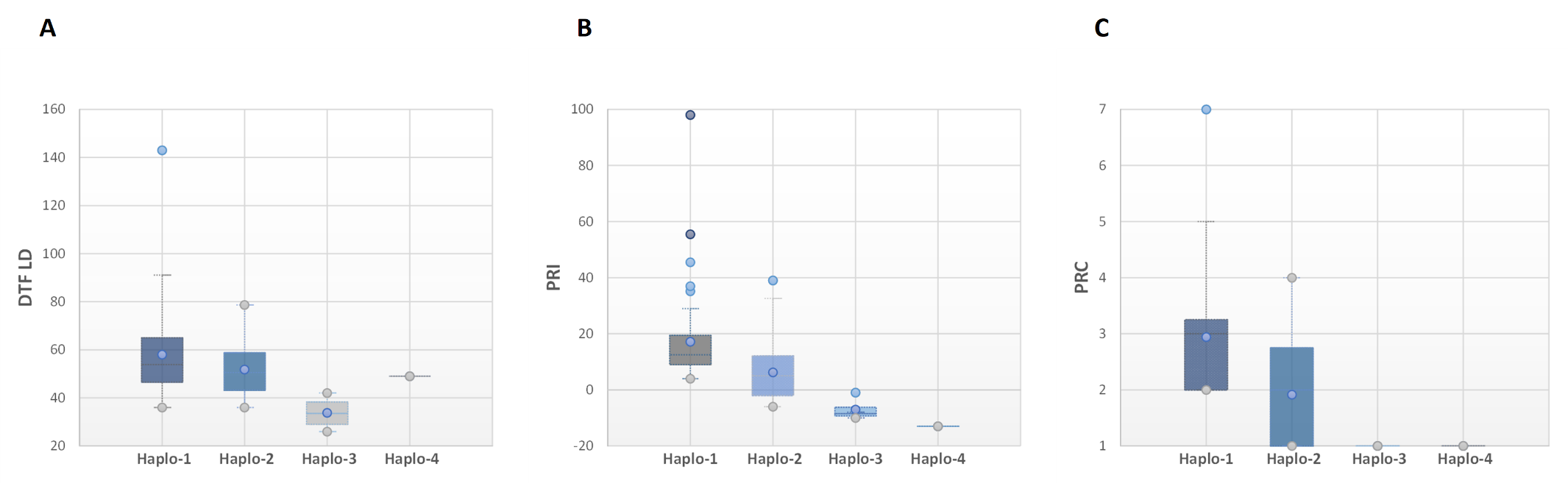


**Figure S8. Distribution of flowering-related traits across *PvE1* haplotypes *in PvCOL2-*deficient accessions.** (**A**) Days to Flowering under long-day (DTF LD)*.* Boxplots show haplotype-specific variation in flowering time. Haplotypes 3 and 4 flower earlier than haplotypes 1 and 2. (**B**) Photoperiod Response Index (PRI = DTF_LD – DTF_SD). Haplotypes carrying non-functional *E1* alleles show PRI values close to or below zero, indicating reduced or absent photoperiod sensitivity. (**C**) Photoperiod Response Classification (PRC). Accessions with functional *E1* alleles are distributed across Neutral, Intermediate, and Sensitive classes, whereas accessions carrying non-functional *E1* alleles (haplotypes 3 and 4) are exclusively classified as Neutral (PRC = 1).


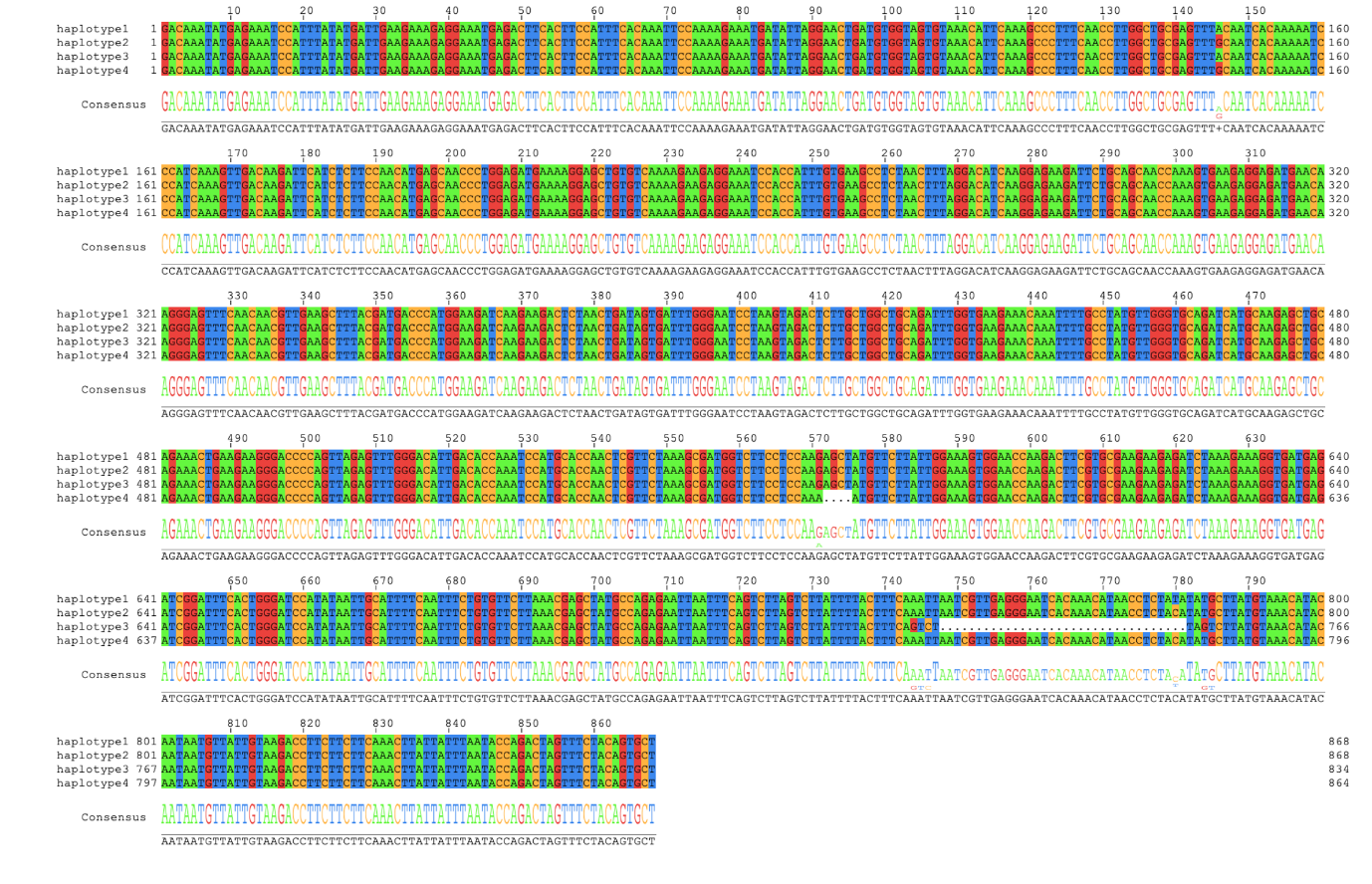


**Figure S9.** **Nucleotide sequence alignment of the *PvE1* genomic region across four haplotypes.** Multiple-sequence alignment of the *PvE1* genomic region (CDS plus UTRs) corresponding to the four haplotypes shown in Figure 7A. Haplotype 3 (*e1-del*) retains the wild-type coding sequence but lacks 34 bp in the 3′ UTR, whereas haplotype 4 (*e1-fs*) is characterized by a 4-bp deletion within the coding region that causes a frameshift at codon 127 and introduces a premature stop codon.
